# Potential candidate genes influencing meat production phenotypic traits in sheep: a review

**DOI:** 10.3389/fvets.2025.1616533

**Published:** 2025-07-16

**Authors:** Ying Han, Muhammad Faheem Akhtar, Wenting Chen, Xiaotong Liu, Mingyue Zhao, Limeng Shi, Muhammad Zahoor Khan, Changfa Wang

**Affiliations:** College of Agriculture and Biology, Liaocheng University, Liaocheng, China

**Keywords:** meat production, carcass weight, vertebral traits, small ruminants, genetic markers

## Abstract

This review examines the genetic basis of meat production phenotypic traits in sheep, addressing the challenge of enhancing carcass and meat quality to meet global demand. The article identifies key potential genes associated with vertebral traits, body size, muscle development, and fat deposition across diverse sheep breeds worldwide. Through comprehensive analysis of recent literature (2018–2025), the study synthesizes findings from genome-wide association studies, candidate gene approaches, and transcriptomic analyses. Specific potential genes like *VRTN, NR6A1, MSTN, ADIPOQ, LCORL, MEF2B, FASN, FABP4, SCD, DGAT1, BMP* and *HOX* family genes demonstrate significant associations with economically valuable traits. The potential genes influencing meat production phenotypic traits (intramuscular fat contents, growth, vertebral traits and body size traits) have been highlighted in this review. This comprehensive genetic marker catalog serves as a critical resource repository for implementing marker-assisted selection programs, providing breeders and researchers with validated genetic targets to accelerate breeding efficiency and enhance meat production in sheep worldwide.

## 1 Introduction

Sheep farming plays a critical role in global agricultural production, serving as a significant source of meat, wool, and other essential products (1). Global food consumption is projected to witness a substantial rise by 2050, particularly in the demand for animal protein products. This demand, however, will not only be driven by quantity but also by the quality of animal protein products desired by consumers. Notably, tenderness stands out as a paramount sensory attribute for consumers when it comes to meat consumption (2, 3). As global food demand continues to rise, there is an increasing imperative to enhance livestock productivity through advanced genetic approaches. The genetic improvement of meat production traits in sheep represents a crucial strategy for addressing these challenges, offering the potential to develop more efficient, high-quality meat-producing breeds that can contribute to global food security.

The complex nature of meat production traits in sheep involves multiple genetic and environmental factors that influence characteristics such as muscle growth, carcass quality, fat deposition, and overall meat quantity and quality (4, 5). Recent advances in molecular genetics and genomic technologies have opened unprecedented opportunities for understanding the genetic mechanisms underlying these important phenotypic traits (6–10). Genome-wide association studies (GWAS), transcriptome and candidate gene approaches have increasingly revealed the intricate genetic architecture that controls meat production characteristics, providing researchers and animal breeders with valuable insights into potential genetic markers and selection strategies (11).

This review article aims to comprehensively explore the current landscape of genetic research related to meat production traits in sheep. By systematically examining recent scientific literature, we will synthesize the most significant candidate genes associated with critical meat production phenotypes across various sheep breeds worldwide. Our analysis will not only highlight the genetic diversity and potential for genetic improvement but also provide a roadmap for future marker-assisted selection (MAS) programs. Through this comprehensive review, we seek to contribute to the ongoing efforts to optimize sheep breeding strategies, ultimately supporting more sustainable and productive livestock farming practices.

## 2 Literature search and selection criteria

This review article was designed to overview the potential candidate genes linked to various meat production traits in sheep. For this purpose, we selected articles published within the last 5 years (2018–2025), reflecting the contemporary landscape of research in the field. However, for the introductory section of this review, we extended our purview to include articles dating back to the year 2015. This comprehensive approach allowed us to establish a robust historical context for the subject matter.

The keywords employed in our search strategy were thoughtfully chosen to capture the multifaceted dimensions of the topic. These keywords included “carcass weight,” “muscle pH,” “muscle tenderness,” “meat quality and quantity,” “vertebrae,” “body size,” “body weight,” “Sheep “molecular breeding,” and “genetic markers, potential genes.” The selection of genes reported by any article for inclusion in this review was underpinned by their recognition as significant (*p < 0.05*) potential candidate genes associated with meat quality and quality-related traits. This recognition was based on the declarations made by authors in their respective published articles, signifying the genes’ significance in the field. To perform functional enrichment analysis and identify biological pathways associated with the genes examined in this review, we used ShinyGO online software (12).

**Figure 1:**
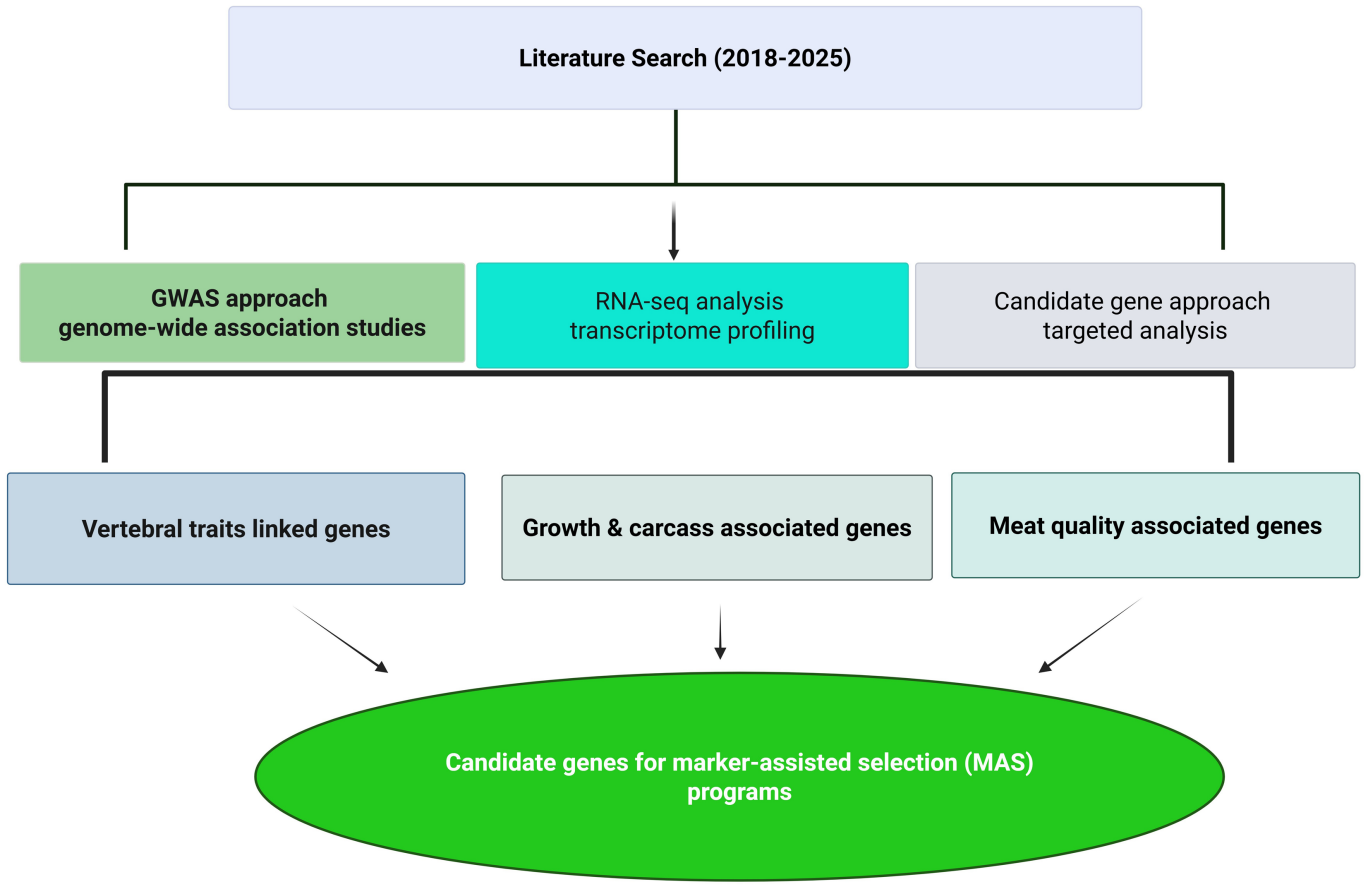
Schematic methodological framework showing the literature strategy and the three main approaches (GWAS, RNA-seq analysis, and candidate gene approaches) used to identify candidate genes for meat production traits in sheep.

In order to maintain a rigorous standard, we excluded articles published in non-science citation index–(SCI) journals and those not published in the English language. This deliberate choice was made to ensure that the articles included in our review were subjected to peer-review processes and accessible to a wider academic audience. Furthermore, it is important to note that book chapters and unpublished data were excluded from our discussion. However, we did consider the foundational insights from previously published review articles pertaining to specific genes associated with meat production traits in small ruminants. The summary of articles used in the current review is provided in Figure 1.

## 3 Overview of potential genes associated with meat production phenotypic traits in sheep

The study of genes associated with meat production phenotypic traits in sheep has significant agricultural and economic importance, as identifying these genetic markers enables more efficient selective breeding programs that can improve meat quality, yield, and production efficiency. Consistently, the association of genes associated with meat production traits have already been documented in previous studies (13, 14). By understanding the genetic basis of traits like muscle growth, fat deposition, tenderness, and flavor profile, researchers can develop genomic-based selection tools that allow producers to make breeding decisions earlier in an animal’s life, reducing costs while increasing genetic gains. Additionally, this genetic knowledge helps address consumer demands for consistent, high-quality meat products while potentially improving animal welfare through selecting for traits that enhance health and reduce stress susceptibility. Such research also contributes to broader food security goals by helping develop more efficient and sustainable sheep production systems that can adapt to changing environmental conditions and market demands.

### 3.1 Potential genes associated with number of vertebral traits in sheep

During the course of livestock evolution, there has been significant variation in the body size of domestic animals, both between and within species or breeds. Among the traits of economic importance, the number of vertebrae is noteworthy due to its association with body length and carcass characteristics. Notably, the association of variations in the number of thoracic and lumbar vertebrae thoracolumbar vertebrae with carcass length have been observed across different breeds of pigs (15), donkey (16–20), sheep (21) and cattle (22). It is worth mentioning that variations in the number of thoracolumbar vertebrae have been considered a selection trait in commercial animal breeding due to its correlations with growth and meat production.

In a general context, the arrangement of vertebrae in sheep typically includes 7 cervical vertebrae (C), 13 thoracic vertebrae (T), 6 lumbar vertebrae (L), and 4 sacral vertebrae (S), resulting in a total of 30 vertebrae. Among these, mutations in the thoracolumbar region, such as T14L6 or T13L7, have been reported as the most common (23). Multi-vertebrae sheep, exhibiting such mutations, demonstrate advantages in terms of adaptability and meat production performance (23). In the case of Kazakh sheep, which are indigenous to west Xinjiang of China, it is observed that there is variation in the number of lumbar vertebrae. Typically, for most sheep, the count includes 13 thoracic vertebrae and 6 lumbar vertebrae, often labeled as T13L6. However, in the case of Kazakh sheep, variations have been found, specifically T13L7 and T14L6, which, respectively, result in increased carcass length by 2.22 cm and 2.93 cm compared to normal T13L6 Kazakh sheep. Additionally, carcass weight is raised by 1.68 kg and 1.90 kg, respectively (23–26). Given the significant economic and productive advantages associated with vertebral variations in sheep, particularly the increased carcass length and weight observed in T13L7 and T14L6 configurations, understanding the underlying genetic mechanisms controlling these traits has become a priority in livestock genomics research. Recent advances in genomic technologies, have enabled researchers to identify candidate genes associated (*SYNDIG1L, VRTN, NR6A1, LTBP2, BMP4*) with vertebral development and segmentation (23, 26–30). Table 1 presents a comprehensive overview of genes associated with vertebral development and bone formation in various sheep breeds. This research area is particularly significant for the sheep industry as the number and structure of vertebrae directly influence carcass length, meat yield, and overall productivity.

**Table 1:** Potential genes associated with number of vertebrae and bone development in sheep.

Genes	Polymorphism	Associated traits	Breeds	Country	Reference
*SYNDIG1L*	g.82573325C > A	Associated with different thoracic vertebral number	Han sheep Sunite sheep	China	(23)
*VRTN*	rs426367238	Correlated with thoracic vertebral number carcass length and carcass weight	China Kazakh sheep	China	(26)
*VRTN, SYNDIG1L, LTBP2* *ABCD4*	rs89393414 C > T	Number of vertebrae	Ujimqin Sheep	China	(27)
*VRTN, NR6A1, SYNDIG1*		Number of vertebrae	Mongolian sheep	China	(28)
*NR6A1*	IVS8-281G > A	Variation of lumbar spine number	Xinjiang Kazakh sheep	China	(29)
*ALX4, HOXB13 BMP4* *EYA2 SULF2*		Embryonic development of tendons, bones and cartilages Development of limbs and skeleton, and tail formation	Ethiopian indigenous sheep	Ethopia	(30)
*SFRP4*	rs600370085: C > T rs415133338: A > G	Associated with bone development Linked with multi-lumbar vertebrae	Duolang sheep	China	(40)
*NID2, ACAN*		Skeletal development and cartilage structure	Afghani sheep	Iran	(41)
*TBXT*		Linked with the caudal vertebrae number and tail length	Sheep	China	(42)
*MGAT4A, KCNH1* *CPOX* *CPQ*	*g.108,610,918C > G* *g.75,716,237C > G* *g.178,730,623 T > G* g.88,323,841 A > G	Number of ribs	Hu sheep (36)	China	(43)
*LTBP2, SYNDIG1L*		Number of ribs and vertebrae	Large fat-tailed sheep, Altay sheep, Tibetan sheep/Ovine Infinium HD SNP BeadChip	China	(44)
*VRTN*, *HoxA*		Linked with vertebral development and associated with thoracic vertebrae Regulates spinal development and morphology	Xinjiang Kazakh sheep	China	(45)
*NDRG2*		Associated with development of the spine Provides valuable resources for the transcriptome of multiple vertebral traits in sheep	Kazakh sheep	China	(46)

### 3.2 Screening potential genes associated with growth, carcass and body size traits using RNA sequencing (RNA-seq) and GWAS in sheep

The integration of RNA-seq and GWAS represents a powerful approach for identifying genes and genetic variants associated with economically important meat production traits in sheep. This comprehensive strategy combines transcriptomic profiling to reveal differentially expressed genes in relevant tissues with population-based association analyses to pinpoint significant genetic variants. By correlating expression pat-terns with phenotypic data and genetic polymorphisms, researchers can identify candidate genes influencing key traits such as muscle growth, fat deposition, meat quality, and carcass composition. Understanding the genetic basis of growth and carcass-related traits in sheep plays a pivotal role in enhancing muscle growth, hypertrophy, and, ultimately, meat production (31, 32). Recently, several meat production associated genetic markers have been identified in various meat sheep breeds (Uruguayan Merino sheep, Romney, Karachaevsky Sheep, Hu, Dorper, Awassi, Afghani, Bandur, Baluch etc.) (Table 2). Consistently, a study has highlighted several genes (*LHX3, LHX4, CAPN, MEF2B, TRHDE, MEF2A, MEF2C, MEF2D, FTO, APOBR, TP53, DRB1 2001, MSTN, GH, GRM1, MBD5, UBR2, RPL7, SMC2,* and *SHISA9*) associated with various meat quality traits, including body weight, growth, and chest girth in sheep (33). Additionally, this study identified genes (*CAST, LEP, MSTN, RFXANK, RIPK2, DGAT1, UCP1,* and *MCPs*) linked to carcass and fat traits in sheep. The genetic analysis of sheep from Table 2 reveals a comprehensive landscape of genes controlling economically valuable production traits. The myostatin gene (*MSTN*) emerges as a critical regulator of muscle development, while *LCORL* and *NCAPG* appear repeatedly as major determinants of growth and body size traits. Fat metabolism and deposition are primarily influenced by *DGAT2, FABP4,* and *SCD,* which regulate lipid biosynthesis and transport. The bone morphogenetic protein (BMP) family, particularly *BMP2,* plays a significant role in both skeletal development and fat deposition in tail regions. Growth hormone pathways involving *GHR* and *IGF1* control overall growth performance, while muscle-specific genes like *MYL2* and *TNNC2* influence meat quality characteristics. Notably, these candidate genes have been validated across multiple sheep populations worldwide using both GWAS and RNA-seq approaches, providing robust genetic markers that could be incorporated into breeding programs aimed at enhancing meat production efficiency and quality in commercial sheep operations. The summary of potential genes affecting meat production phenotypic traits in sheep is provided in Table 2.

**Table 2:** Potential genes associated with growth, carcass and body confirmation phenotypic traits using GWAS and RNA-seq in sheep.

Genes	Associated traits	Breeds/methods	Country	Reference
*ELOVL2, ARAP2, IBN2, TPM2*	Associated with meat production traits (muscle contraction and fatty acid composition)	Colombian Creole hair sheep	Colombia	(5)
*NPR2, HINT2, SPAG8, INSR, FABP3, DIS3L2*	Body size traits (body weight and height), growth traits and fat deposition	Ethiopian indigenous sheep/Illumina Ovine 50 K SNP BeadChip assay/GWAS	Ethopia	(30)
*SLC9C1, VSTM2A, FRG1*	Body size traits (chest girth, cannon circumference, hip width, body height, and body length)	Hulunbuir sheep/GWAS	China	(47)
*ACLY, SLC27A2, COL1A1, HOXA9, PGM2L1, ABAT*	Faster growth, fat deposition and muscle development	Sheep/Meta-analysis	China	(48)
*CRADD, HMGA2, MSRB3, PTCH1, MSTN, PDE3A, LGALS12, GGPS1, SAR1B*	Growth rate Body size Muscle development and fat metabolism	10 Chinese indigenous breeds 5 Western sheep breeds	China	(49)
*IGFBP6, ST7, SCD5, DTNBP1, OAR2, KYNU, FGF12, FTO*	Live weight, bicoastal diameter, rump width, heart girth, cannon bone circumference.	Saryarka/GWAS	Kazakhstan	(50)
*ARHGAP31, EPS8, AKT3, EPN1, PACS2, KIF1C, FSTL1, PTGFRN, IFIH1*	growth and slaughter performance	Hu sheep	China	(51)
*CAMK2B, CACNA2D1, CACNA1C, FGF9, BMPR1B, FIGF, WT1, KCNIP4, JAK2, WWP1, PLCL1, GPRIN3, CCSER1*	Birth weight Weaning weight Monthly related weight	Hu sheep	Iran	(52)
*ASB3, THADA, PRPS1L1, MTHFS, RALGAPA1,MEIS1, AKIRIN1, GRXCR1, ANKS1B, CFI, SLCO2B1, KRAB*	Meat production traits	Merino/Ovine Infinium HD BeadChip 600 K/GWAS	Russia	(53)
*CLVS1, EVC2, KIF13B, KCNH5, NEDD4, LUZP2,* *MREG, KRT20, KRT23, MSTN, MEF2B, FABP4, FZD6*	Meat production traits	Karachaevsky Sheep/Ovine Infinium HD BeadChip 600 K/GWAS	Russia	(54)
*CAST, LAP3, MED28, HERC6, CDH10, TMC2, SIRPA, CPXM1*	Live weight and body condition score	Uruguayan Merino sheep/GWAS	Newzealand	(55)
*LEKR1*, *LCORL*, *GHR*, *RBPJ*, *BMPR1B*, *PPARGC1A*, *PRKAA1*	Growth traits	Merino/Ovine SNP50 BeadChip	Italy	(56)
*CYP7B1*	Body condition score and live weight	Rasa Aragonesa/HD Illumina Ovine BeadChip	Spain	(57)
*BMPR1B*, *HSD17B3*, *TMEM63C*	Body weight traits	Qira Black sheep German Merino sheep	China	(58)
*ACTA1*, *MYH11*, *WAS*, *VAV1*, *FN1*, *ROCK2*	Muscle development	Han and Tan Sheep/Whole-genome bisulfite sequencing (WGBS)	China	(59)
*KDM4C*, *TGFB2*, *GOT2, HILPDA*, *FAT1, MMP12*, *MMP13*	Meat quality traits	Texel Sheep × Altay Sheep/Ovine SNP 600 K BeadChip/GWAS	China	(60)
*TNC*, *TNFSF8*, *COL28A1*	Extracellular organization, live weight	Romney ewe lambs	New Zealand	(61)
*OLFML3*, *ANGPTL2*, *THOC5*	Muscle tenderness	Garut composite sheep	Indonesia	(62)
*AM184B*, *NCAPG*, *MACF1*, *ANKRD44*, *DCAF16*, *FUK, LCORL*, *SYN3*	Live weight, growth of muscle and bone	Alpine Merino Sheep/Sheep 50 K Panel/GWAS	China	(63)
*RALYL, POM121C*, *PHIP*, *ZIM3*, *PEG3*, *TRPC7*, *FBXL4*, *DNAAF2*	Carcass traits (*Longissimus dorsi* muscle depth and back-fat thickness)	Esme sheep	Turkey	(64)
*DGKB, PAK1, CTTNBP2, CHL1, NALCN*, *NFATC2*	Meat quality traits	Caucasian sheep	Russia	(65)
*DLK1, MYOD1, GH, REM1, MF2B*	Meat productivity traits	Jaglin Sheep	Russia	(66)
*ALS2*, *ST6GAL2*, *PLXNA4*, *DPP6*, *COL12A1*	Carcass traits (rib eye muscle)	Hu Sheep	China	(67)
*TGFB1*, *TGFB3*, *FABP3*, *LPL*	Growth and development	Dorper sheep	China	(68)
*TLE4, MYOM3, SLC44A1, TMEM50A*	Growth trait	Akkaraman sheep	Turkey	(69)
*MYLK3*, *MYL10*, *FIGN*, *MYOM3*, *LMCD1*, *FLRT1*, *MYHs*	Muscle growth and development, fat deposition in muscle	Southdown × Hu, Suffolk × Hu, Hu × Hu/RNA-sequencing	China	(70)
*FAIM, MRAS, PIK3CB, NHLH2, CASQ2, GLIS3, TMOD1, CNTN1, NAALADL2, ATPL1, LRRK2, HMGA2, MSRB3, ANKS1B, IR29A, LCORL, NCAPG, DTHD1, ARAP2, SYNE2, SPTB, KHDRBS3, CLVS1, NKAIN3, UBL3, SLC7A1, GSKIP, BDKRB2, SETD3, BCL11B*, *LRRK1*	Carcass traits	Santa Ines lambs/50 K SNP chip	Brazil	(71)
*SPAST*, *TGFA*, *ADGRL3*	Carcass: external carcass length, leg length, carcass yield, commercial cuts weight, loin eye area and subcutaneous fat thickness	Santa Ines SHEEP/Illumina OvineSNP50 BeadChip array	Brazil	(72)
*LIPE, LEP, ADIPOQ, SCD*, *FASN*	Meat quality (Muscle development, muscle fibre)	Tibetan sheep/RNA-Seq	China	(73)
*MSTN*, *IFRD1*, *PPARD*, *MYL2*	Meat quality and growth	Han sheep/RNA-Seq	China	(74)
*PDGFD*, *FGF18*, *SRF*, *SOCS2, HOXA, BCL2L11*, *TSHR*	Development and growth traits	Luxi Black Head sheep	China	(75)
*BMP2*, *HOXA11*, *PPP1CC*, *LPIN1*	Regulation of adipogenesis Intramuscular fat deposition	Hu sheep and Tibetan sheep	China	(76)
*CERS6*, *BTG1*, *RYR3*, *SLC6A4*, *NNAT*, *OGT*, *SCD5*	Body size and fat deposition	Sheep local breeds/Ovine Infinium HD SNP BeadChip	China	(77)
*CHRNB1*	Live weight	Merino sheep/Illumina Ovine single nucleotide polymorphism (SNP) 54 BeadChip//GWAS	China	(78)
*ZNF704, AK2*, *PARK2, MOCOS*, *ELP2, MFAP1*	Body weight, tail length, chest width and girth	Qira black sheep	China	(79)
*ATP8A2*, *PLXDC2*	Post-weaning weight	Lori-Bakhtiari sheep	Iran	(80)
*PDGFD, BMP2*	Fat deposition on tail	Altay and Tibetan/Illumina Ovine SNP600 BeadChip/GWAS	China	(81)
*MEG8_2, LCORL, DOCK8*, *PGM5*, *DMRT1, SLC16A1, GHR, POLR1B, SHISAL1, LLPH, MASP1, FAM3C*, *WNT16, SYNPO*, *CDX1, PDGFRB*, *SETBP1*	Meat productivity and carcass traits (meat mass and meat fat)	Merino, Poll Dorset, Border Leicester, Suffolk, white Suffolk, Texel, Corriedale, Coopworth	Russia	(82)
*FOXN3*, *CNTN3, FTO*, *CFAP73*, *ARPP21*, *RAB21, RBM45*, *SHC4*, *ADAMTS9*, *FRMPD4*, *ZFP36L1*, *ACTN1*, *ASTN1*	Growth and development of cells and tissues.	Jalgin merino/Ovine Infinium HD BeadChip 600 K/GWAS	Russia	(83)
*MCTP1, COL4A6, CADM2, KITLG*	Chest circumference and body height	Hu sheep/GWAS	China	(84)
*EYA2*, *GDF2*, *GDF10*, *MEF2B*, *SLC16A7*, *TBX15*, *TFAP2B*, *TNNC2*, *CPXM2*, *LRIG3*	Growth traits	Barki sheep/Illumina OvineSNP50 V2 BeadChip//GWAS	Germany	(85)
*APOA5, SLC25A30, GFPT1, LEPR, FABP7, GSTCD, CYP17A, APOA5, CFHR5, TGFBR2*	Fat deposition, Fatty acids composition	Indonesian Javanese thin-tailed sheep/RNA-seq	Indonesia	(86)
*FGFRL1*, *SIX1*, *PLCB1*, *CRYAB*, *MYL2*, *ADIPOQ*, *PPARD*, *IGF1*, *LARGE, GPX1*, *GPC1*	Growth, development, and meat quality	Dorper × Small Tailed Han sheep and Mongolia× Small-tailed Han sheep/RNA-seq	China	(87)
*PAPPA2*, *NR6A1*, *SH3GL3*, *RFX3*, *CAMK4*	Growth, development, body confirmation and carcass traits	snow sheep and argali/GWAS	China	(88)
*ITGA11, SCMH1, CAMTA1, CAPN6*	Birth weight and yearling weight	Hu sheep/GWAS	China	(89)
*CDS2*, *PROKR1, BMP2*	Fat deposition in tail and tail length	Tunisian sheep/GWAS	Tunisia	(90)
*FOSL2, TMEM117, LECT2, TRAK1*	Chest girth, Body length, body weight	Luzhong mutton sheep/ Illumina Ovine SNP50 Bead Chip	China	(91)
*SPARC*, *ACVRL1*, *FNDC5*, *FREM1*	Meat quality traits	Small-tailed Han sheep × Mongolian sheep/RNA-Seq	China	(92)
*AADACL3*, *VGF*, *NPC1*, *SERPINA12*	Birth, weaning, yearling and adult weight	Alpine Merino sheep, Alpine Merino sheep, Aohan, Qinghai wool sheep	China	(93)
*FOXF2*, *MAPK12*, *MAP3K11*, *STRBP*	Body weight traits	Hu sheep/high-density 600 K SNP arrays/GWAS	China	(94)
*DGAT2, ACSL1, ACACA, SCD, ADIPOQ, ACLY, CPT2, ADCY6, FASN, PER3, CSF1R, SLC22A4, GFPT1, CDS2, BMP6, ACSS2, ELOVL6, HOXA10, FABP4*	Fat deposition in tail region	Lori-Bakhtiari and Zel/RNA-sequencing	Iran	(95)
*NOT2*, *CNOT6*, *HSPB1*, *HSPA6*, *MAP3K14, PPARD*,	Development of muscle, intramuscular fat deposition	Bandur sheep/RNA-sequencing	India	(96)
*MURF2*, *FBF1*, *DTNBP1*, *SETD7*, *RBM11*	Body length, body height, chest girth, tail length, tail width, tail circumference, carcass weight, tail fat weight	Hulun Buir sheep/GWAS	China	(97)
*RAB6B, TF, GIGYF2*	Birth weight	Lori-Bakhtiari sheep/Illumina Ovine SNP50 Bead Chip	Iran	(98)
*PDGFRA*, *PDGFD*	Fat deposition	Italian sheep/OvineSNP50K array/GWAS	Italy	(99)
*DGAT2*, *TRHDE*, *TPH2*, *ME1*, *UBE3D*, *PARP14, MRPS30*	Fat composition in Longissimus dorsi muscle	Santa Inês sheep/Ovine SNP50 BeadChip/GWAS	Russia	(100)
*MAGI1*, *ZNF770*	Growth traits	Baluchi sheep/Illumina OvineSNP50 BeadChip/GWAS	Iran	(101)

### 3.3 Candidate gene approach to screen potential genetic markers associated with meat production phenotypic traits in sheep

The candidate gene approach represents a targeted strategy in sheep genetics research that focuses on identifying and analyzing specific genes with potential influence on economically important meat production traits. This method selectively examines genes with known biological functions related to muscle development, growth, fat deposition, and meat quality characteristics based on prior physiological knowledge or findings from other livestock species. For example, researchers typically analyze polymorphisms within these candidate genes—such as myostatin (*MSTN*), calpain (*CAPN*), calpastatin (*CAST*), leptin (*LEP*), *DGAT1* and growth hormone (*GH*)—to establish associations with phenotypic traits including carcass weight, muscle mass, intramuscular fat content, tenderness, and meat flavor profile (Table 3). Consistently, our previously published research extensively examined the role of *DGAT1 K232A* polymorphism in enhancing sheep meat quality traits (34). Fatty acid-binding protein 4 (*FABP4*) is involved in fatty acid transportation, and variations in this gene have been reported to influence fat deposition in mammals. Several studies have consistently demonstrated the involvement of *FABP4* in regulating meat quality traits in sheep (35). Additionally, Alwan et al. (35) observed a detrimental effect of p.61Thr > Asp on *FABP4*, resulting in reduced fatty acid binding efficiency and increased carcass traits in Karakul and Awassi Sheep. Furthermore, other studies have documented associations between *FABP4* variations and various economic traits in sheep, such as carcass and growth traits in New Zealand Romney lambs (36), morphometric traits in Albanian sheep (37), body weight, final weight, and average daily gain in three Egyptian sheep breeds (38), as well as intramuscular and internal fat weight in two Russian sheep breeds (39). The approach has proven valuable for marker-assisted selection programs in sheep breeding, allowing producers to make informed breeding decisions that enhance meat production efficiency and quality while reducing the time and resources required compared to genome-wide studies. Despite limitations in detecting novel genes, the candidate gene approach continues to provide practical applications in sheep breeding programs focused on improving commercially relevant meat production traits. The summary of determinant genes associated with meat production phenotypic traits in sheep is provided in Table 3.

**Table 3:** Potential genes and their polymorphisms associated with growth, carcass and body confirmation phenotypic traits in sheep using candidate gene approach.

Genes	Polymorphism	Associated traits	Breeds/methods	Country	Reference
*MST1, MST2, YAP, MOB1A*		Chest circumference, hip height, body height, body weight, and body length	Tong sheep, Hu sheep, Small Tail Han sheep, and Lanzhou large-tailed sheep	China	(102)
*NSMF*		Cannon circumference	Chaka sheep, Hu sheep and Small-tailed Han sheep	China	(103)
*PDK4*		Intramuscular fat (IMF) content of meat	Han sheep and two cross breeds	China	(104)
*PIK3R1*		IMF deposition	Han sheep	China	(105)
*MSTN*	C2361T	Wider chest, waist, and hip widths t	Charolais sheep, Australian White sheep, crossbreeds of Australian White and Small-tailed Han, and crossbreeds of Charolais and Small-tailed Han	China	(106)
*ADIPOQ*	c.198,473 337C > A	Live body weight and body measurements.	200 Awassi sheep/SSCP	Iraq	(107)
*RETN*	233A > C	Associated with lower body weight and length, chest and abdominal circumferences, and wither and rump heights	190 Karakul and 245 Awassi breeds/	Iraq	(108)
*CDH18*	rs423955510 rs412944692 rs416959317 rs398980439 rs428685044	Growth traits (body weight and body size)	1,008 Hu sheep/Illumina Ovine SNP 50 K BeadChip	China	(109)
*GH*		Growth and carcass traits	Egyptian Awassi sheep/PCR-RFLP	Egypt	(110)
*DDC*	g.5,377,439 G > A	Meat quality and carcass traits	189 Indonesian sheep/PCR-RFLP	Indonesia	(111)
*IGF1*	g.171328230 delT rs401028781 rs422604851 g.171328404C > Y g.171328260G > R g.171328246 T > A g.171328257 T > G g.171328265 T > C	Chest width at weaning and leg circumferences at yearling Higher *Musculus longissimus dorsi*	Kıvırcık, Karacabey Merino, Ramlıç, German Black-Head Mutton × Kıvırcık, Hampshire Down × Merino	Turkey	(112)
*CD8B*	chr3:62,718 030 G > A	body weight, body length	Hu Sheep	China	(113)
*ALB-1*	g.8699 A > T		Hu sheep	China	(114)
*ALB-2*	g.13458 T > C	Body weight			
*AHSG*	g.19484 A > C g.2454 T > C	Body weight, body height, body length			
*NCAPG*	rs424493003 T > A rs159958117 C > T rs423376306 T > C rs417096593 C > T rs430255987 T > A	Growth and myogenic development	Hu sheep	China	(115)
*IGF2BP1*		Growth traits (Body weight)	Hu sheep	China	(116)
*FADS3*	g.2,918 A > C	Body weight, body height, body length, and chest circumference	Hu sheep	China	(117)
*PTPN3*		Growth traits (body weight and body size).	Gansu alpin, Merino,	China	(118)
*PLAG1*	g.8795C > T	Birth and weaning weights	Hu sheep	China	(119)
*HMGA1*	g.5312C > T	Tail fat weight, relative weight of tail fat, and relative weight of tail	Hu sheep	China	(120)
*CAPN3*		Birth weight trait	Merino × Garut (MEGA) backcross sheep	Indonesia	(121)
*PDE2A, ARAP1, PCDH15*		Low meat productivity	Argali, Romanovskaya	Russia	(122)
*HIAT1*	rs1089950828	Growth traits	Luxi black-headed sheep and Guiqian semi-fine wool	China	(123)
*PDGFD*		larger body length, chest depth, and body weight	Luxi black-headed sheep	China	(124)
*PRKAA2*	chr1:32832382 G > A	Body weight, body length, chest circumference, and cannon circumference	Hu sheep and Dorper sheep	China	(125)
*HTR4*	g.101220C > T	Growth traits	Hu sheep	China	(126)
*LRRC8B gene*		Growth traits (chest depth)	Han Sheep	China	(127)
*LRRFIP1*		Heart girth, rump breadth, circumference of the cannon	Hu sheep	China	(128)
*MEF2B*	g0.14327 G > C g0.16706 T > A	Carcass and growth traits	Awassi and Cukurova	Turkey	(129)
*POMC*	rs424417456: C > A	Body weight and length, wither and rump height, chest and abdominal circumference	Karakul and Awassi sheep	Iraq	(107)
*BMPR1B*		Growth traits	MEGA (Merino × Garut)/PCR–RFLP method	Indonesia	(130)
*MAP3K5*	g.205261 A > G	Body height, body length, chest circumference, and cannon circumference	Hu sheep	China	(131)
*PLIN1, FTO*		Body weight, body height, chest width, chest depth, cannon circumference, head length, coccyx length, forehead width, and back height.	Hu, Dupor and Han sheep	China	(132)
*KAT6A*		Body confirmation traits (body length)	Small-tailed Han, Chaka and Hu sheep	China	(133)
*CAST*	c.1210C > T c.646G > C c.1437G > A c.2097C > T	Fatty acid composition in meat and meat quality	Sonid sheep	China	(134)
*CTSK*	g.106510225G > A	Average daily weight gain, fat-tail weight to carcass weight ratio, muscle thickness and muscle cross-sectional area	Afshari × Booroola-Merino crossbred sheep/SSCP-PCR	Iran	(135)
*FST*	g.25634085C/C	Body size traits	Iranian Mehraban sheep/SSCP-PCR	Iran	(136)
*METTL21C, PPARGC1A, WFIKKN2*		Associated with carnosine, a metabolite related to meat quality Muscle growth and development	Hu sheep	China	(137)
*STAT3*		Body height and rump width in Hu sheep Body length in Tong sheep	Han, Tong and Hu sheep	China	(138)
*JAK1*		Body height, body oblique length and cross height in Hu sheep Body oblique length and cross height in Han sheep			
*GH, DGAT1*		Body weight and tail length	Awassi sheep/PCR-RFLP	Turkey	(139)
*IGF-I, IGFALS*		Growth traits (birth weight)	Hamdani sheep	Turkey	(140)
*MSTN, CAST*		Growth traits (body weight, body length, chest depth, heart girth and withers height)	Awassi sheep	Turkey	(141)
*ETAA1*		Growth traits	Luxi Blackhead sheep, Lanzhou fat-tailed sheep, Hu sheep, Tong sheep, and Tan sheep	China	(142)
*OLFML3*	g.90317673C > T	Meat quality traits (tenderness and cooking loss), carcass characteristics (carcass length), retail meat (pelvic fat in leg, intramuscular fat in loin and tenderloin, muscle in flank and shank; fatty acids composition)	Javanese, Garut, Barbados, Compass agrinak, Jonggol	Indonesia	(143)
*IGF1* *IGF1R*	rs600896367 rs600896367 rs400398060 rs162159917	Growth traits	Hulun Buir	China	(144)
*DGAT1*	K232A	Associated with increase loin meat yield	Romney, Coopworth, Perendale, Corriedale, Merino, Texel, Suffolk, Southdown, Poll Dorset, and Borderdale	New Zealand	(145)
*IGF-1R*		Longissimus dorsi (LD) muscle depth, skin thickness, and fat thickness, muscle development, borth weight, daily weight gain	Turkey local sheep breed	Turkey	(146)
*CTNNA3*	g.2018018 A > G	Body weight, body height, body length and chest circumference	Hu sheep	China	(147)
*CAP2*	g.8588 T > C	Body height			
*FASN* *ELOVL5*	g.12694 A > G g.62534C > T	Reduce fat deposition in tail region	Sheep	China	(148)
*HOXB13*		Tail length	Merinolandschaf	China, Germany	(149, 150)
*PLAG1*		Growth traits	Luxi Blackhead sheep	China	(151)
*MYF5*	g.6838G > A g.6989 G > T g.7117C > A g.9471 T > G	Body weight, body length, withers height, chest depth, chest circumference, chest width, cannon bone circumference and hip width	Grassland short-tailed sheep		(152)
*GHR*		Body weight, body height, chest depth, chest width, chest circumference, cannon circumference, paunch girth and hip width	Luxi Blackhead sheep	China	(153)
*KLF15*	c.62565119 A > G	Body weight, body height, and body length	Hu sheep	China	(154)
*GHE5*	c.1588C > Y(C/T) (Ala160Val), c.1603A > M(A/C) c.1604G > S(G/C) (Lys165Thr), c.1606A > W(A/T) (Gln166Leu), c.1664C > Y(C/T)	Longer body length, wider leg circumferences, and thinner cannon bone perimeter, greater percentage of neck, shoulder, and leg, greater percentage of loin, and a greater percentage of rack	Kıvırcık, Karacabey Merino, Ramlıç, German Black-Head Mutton × Kıvırcık, Hampshire Down × Merino crossbreed/SSCP	Turkey	(155)
*PPARGC1A*		Growth traits (Body weight and height) and fat deposition in muscle	Hu and Grassland short-tailed sheep	China	(156)
*TRAPPC9* *BAIAP2*	g.57654 A > G g.46061C > T	Weight of tail fat, tail fat relative weight (body weight), and tail fat relative weight (carcass)	Hu sheep	China	(157)
*RAP1GAP* *rBAT*	g.13561 G > A g.1460 T > C	Tail width, tail fat weight and relative tail fat weight	Hu sheep	China	(158)
*CAPN, CAST, LEP, GH, IGF-1*		Birth weight, body back fat thickness, muscle development	Merina sheep	Colombia	(159)
*CLPG*		Carcass weight, growth and meat quality	Kıvırcık, Karacabey Merino, Ramlıç, German Black-Head Mutton × Kıvırcık, Hampshire Down × Merino	Turkey	(160)
*FTO*	23704451C > A	Tail length and the weight of tail fat	Hu sheep	China	(161)
*TOP2B*		Body height, height of hip cross, chest and canon circumference,	Chaka sheep, Hu sheep, Small-tailed Han sheep	China	(162)
*MyoD1, MyoG, MSTN*		physicochemical meat traits (Muscle tenderness, pH)	Santa Inês sheep	Brazil	(163)
*SSTR5*	rs601836309 rs400914340 rs413380618 rs605867745	Body weight, body height, body length, chest circumference, chest depth, chest width, hip width, and cannon circumference	Hulun Buir sheep	China	(164)
*PPARGC1B,* *ZEB2*	g.300 G > A g.645C > T	Body weight traits	Hu sheep	China	(165)
*BAG4*		Body height, body slanting length, body height and hip cross height	Chaka, Hu sheep and Small Tail Han sheep	China	(166)
*HSL* *LEPR*	c.1865C > T c.2038 T > C c.2800G > A c.2978C > G	Birth weight, weaning weight, marketing weight	Barki lambs/SSCP/PCR	Egypt	(167)
*PRL*		Body weight, body height	Luxi Blackhead sheep	China	(168)
*CREB1*		Body length, height, and index; chest width, depth, and width index; cannon circumference index; and height at the hip cross	Mongolian sheep	China	(169)
*GnRH1*	5′-UTR:50 A > C intron1:264 G > C	Growth traits	Awassi (123) Karakul (78)/PCR-SSCP	Iraq	(170)
*GLIS1*	g.27807636G > T	fat deposition in sheep tails	Mongolian and Small Tail Han sheep	China	(171)
*SCD* *FABP4* *FASN*	*g.12323864A > G* *g.62829478A > T* *g.12323864A > G*	Meat quality traits including IMF, long-chain polyunsaturated fatty acids (LC-PUFA), and functional meat products (FMP)	Tattykeel Australian Whit	Australia	(172)
*FTO*		Partial growth traits, tail length, and fat deposition on tail	Tong sheep	China	(173)
*IGF1R*	c.654G > A	Cold carcass, leg part, leg cut, fore shank, and kidney weights, as well as eye of loin depth, IMF content, and water -holding capacity of meat	Colored Polish Merino sheep/PCR-SSCP	Poland	(174)
*FGF5*	g.105922244 A > G g.105922334 A > T g.105922340 G > T g.105922232 T > C g.105914953 G > A	Body weight and height	South African mutton merino (♂) × Gansu alpine fine wool (♀)	China	(175)
*MEF2B* *UCP3*	g.1826C > T g.10266 G > C	Average body weight and chest and cannon circumference	Hu sheep	China	(176)
*FAM184B*		Body composition and fatty acid contents in muscles	Merino and Coopworth	Australia	(177)
*IGF1*		Hot carcass weight, carcass fat depth at the 12th rib	Romney sheep/PCR-SSCP	Newzealand	(178)
*STAT3*		Body weight and fatness traits	Hu sheep	China	(179)
*SSTR1*	C309T (rs404696179) A285G (rs426187704)	Body weight, body height, body length, chest circumference, chest depth, chest width, hip width, and cannon circumference	Hulun Buir sheep	China	(180)
*CYP2E1*	g.50657948 T > G	Meat and tenderness, as well as fatty acid composition	Javanese fat-tail, Javanese thin-tail, Garut, Jonggol, compass agrinac, Barbados/PCR-RFLP	Indonesia	(181)
*CYP17*		Growth traits	Sheep	Turkey	(182)
*MSTN*	c.159 A > T c.173 T > G	Birth weight and average daily weight gain	Barki, Rahmani, Ossimi, Saudi Arabian Najdi	Iran	(183)
*PIGY*		Body weight, chest circumference, and tube circumference	Han, Hu, Chaka/PCR-SSCP	China	(184)
*LEP*	rs420693815	Weaning weight and average daily gain	Barki sheep	China	(185)
*KMT2D*		Body length, withers height, hip width	Han, Hu, Chaka	China	(186)
*PDGFD*		Fat deposition in tail region	Sheep	China	(187)
*GHR, GHRH, GHRHR*		Higher hip height, reduced body height chest depth, hip width and cannon girth	Han, Tong, Lanzhou fat-tail	China	(188)
*IGFALS*		Chest girth, weaning weight, body weight	Ghezel and Makouei	Iran	(189)
*CHCHD7*		Growth and development traits (body length, chest depth and chest width)	Tan, Luxi Blackhead, Small-Tail Han, and Lanzhou Fat-Tail sheep	China	(190)
*LPIN1*		Decrease in birth weight and the proportion of leg yield, but with an increase in hot carcass weight and the proportion of loin yield. Increased pre-weaning growth rate and shoulder yield	Romney/PCR-SSCP	New Zealand-China	(191)
*PDGFD*		Regulation of adipogenesis and fat deposition in tail region	Thin sheep breeds/Illumina Ovine 50 K Beadchip	China	(192)
*BMP2*	g.48401272C > A g.48401136C > T	Fat deposition in tail	Tibetan and Hu sheep	China	(193)
*LIPE*	g.151C > A g.198C > T/exon 2, g.213G > C g.226G > T g.232A > C/exon 9	Higher dressing percentage and lower fat tail weight	Awassi/PCR-SSCP	Iraq	(194)
*RAB44*		Growth and meat/carcass traits,	Blackhead Persian, Nguni and Namaqua Afrikaner	South Africa	(195)
*SIRT7*		Body size traits (Rump width, chest depth)	Lanzhou fat-tail sheep and small-tail Han sheep	China	(196)
*ZNF395*		Chest width and circumference in Han sheep, cannon circumference in Hu sheep, fat deposition in tail in Lanzhou sheep	Hu, Han and Lanzhou sheep	China	(197)
*CAST*		Final body weight and longissimus muscle width	Awassi sheep	Jordan	(198)
*IGF1*	rs430457475 rs412470350 rs409110739 rs400113576	Internal carcass length, rump girth, rib yield and neck weight, rib weight, rib yield, loin weight, loin yield, leg weight, neck weight and carcass finishing score	Santa Ines sheep/PCR-SSCP	Brazil	(199)
*PROP1*		Higher lamb tailing and weaning weights, and growth rate-to-weaning	Romney sheep	New Zealand	(200)
*MSTN*	c.1232G > A	Body weight	Kamieniec sheep/PCR-SSCP	Poland	(201)
*ACACA, NCAPG, LCORL*		Carcass and growth traits (body weight, post-weaning gain, bone-related traits, muscle depth, fatty acid formation)	Based on meta-analysis in various sheep breed	Russia	(202)
*MSTN*	c.1232G > A	Carcass quality, meat quality, and biometric traits	Polish Merino sheep/PCR-SSCP	Poland	(203)
*LCORL, SPP1,* *LAP3, LCORL*		Birth weight and yearling weight	Hu sheep	China	(204)
*CAST*		Intramuscular fat deposition	Polish Lowland Sheep, Finnsheep or Romanov, Suffolk, Charolaise	Poland	(205)
*MC4R*	−103C > G −206G > A −943G > T −1026G > A	Birth weight, weaning weight, and backfat thickness	Hu sheep	China	(206)
*ORMDL1*		Body weight, body height, body length, chest depth, and height of hip cross in Han sheep, Body height, heart girth, and circumference of cannon bone in HU sheep	Small-tailed Han sheep Large-tailed Han sheep Chaka sheep Hu sheep	China	(207)
*BMP4*		Post-weaning daily gain, marketing weight, height at hips, thigh circumference, body mass index and skeletal muscle index.	Barki lamb/PCR-SSCP	Egypt	(208)
*PITX2*		Chest width, hip width, chest depth, chest circumference, and body height,	Hu sheep, small-tailed Han, Tong, and Lanzhou fat-tailed sheep	China	(209)
*PPARGC1A*		Valuable cuts weight, hot carcass weight and carcass fatness	Texel sheep	Uruguay	(210)
*DGAT1*		Live weights, fat thickness, rib-eye area and shoulder weight	Texel sheep Tong, Small Tail Han and Hu sheep	Uruguay China	(211)
*GHR*		Birth weight and carcass fatness			
*GHRHR*		Live weights and fat thickness			
*PRNP*		Growth traits, chest width in Small Tail Han sheep, chest circumference in Hu sheep, tail length in Tong sheep			
*SSTR1*		Birth weight, weaning weight, pre-weaning growth rate, hot carcass weight, subcutaneous fat depth, leg, loin, shoulder and total lean meat yield	Romney lambs/SSCP-PCR	China-New Zealand	(212)
*APOA5*	g.26929941C > T	Polyunsaturated fatty acids and fat deposition in muscle	Javanese Fat Tailed, Javanese Thin Tailed, Garut Composite Sheep	Indonesia/SSCP-PCR	(213)
*UCP1*		Decreased hot carcass weight, loin lean-meat yield, leg lean-meat yield in the carcasses	Romney lambs/SSCP-PCR	China-New Zealand	(214)
*LEP*	g.92501372 G > A g.92501407C > T g.92501543 A > G g.92503024 G > A	Neck weight and neck yield, hot and cold carcass weights, leg yield, internal carcass length and carcass finishing	Santa Ines sheep	Brazil	(215)

## 4 Discussion

**Figure 2:**
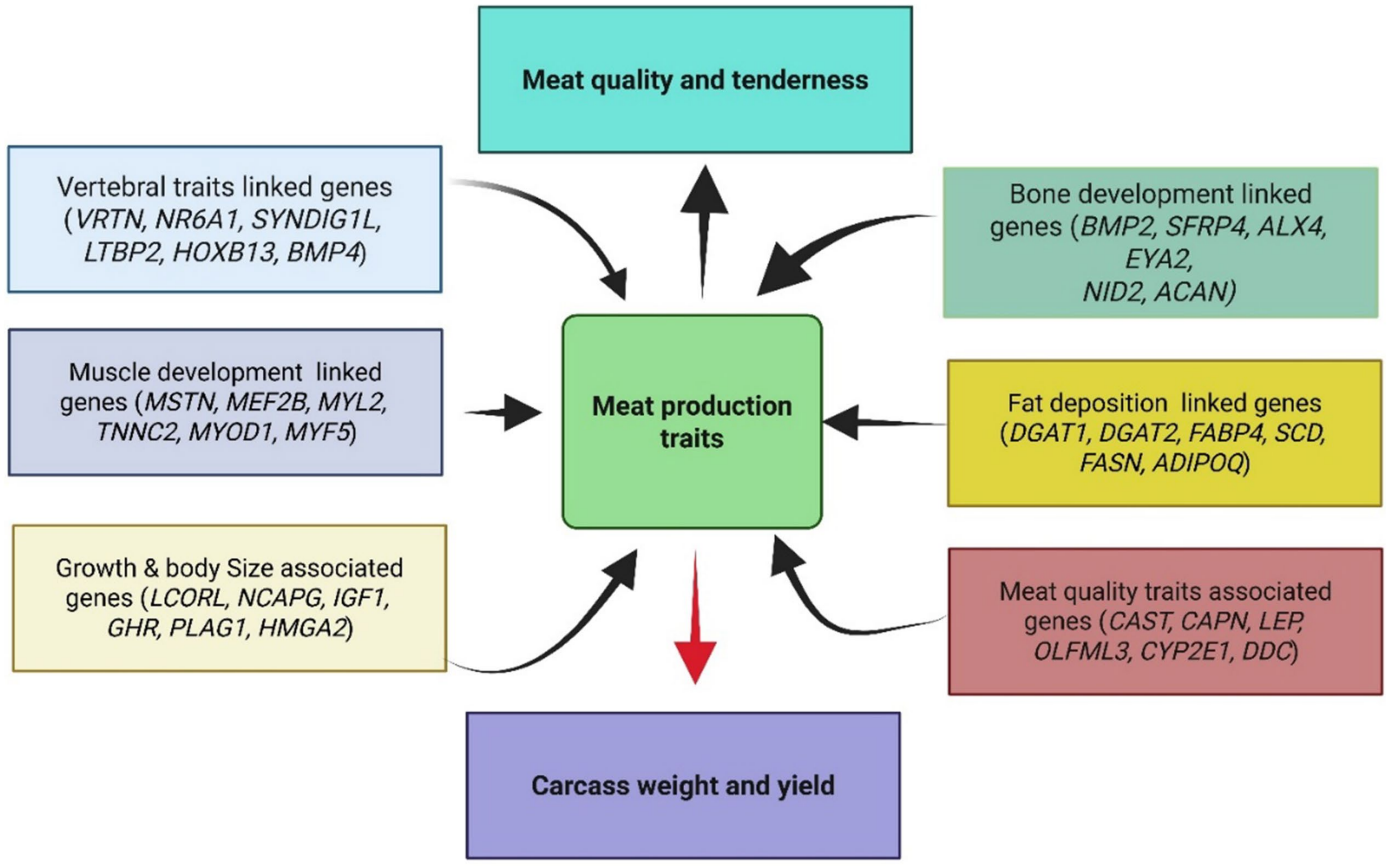
Conceptual framework showing the major gene categories affecting meat production traits in sheep. Genes are grouped by their primary biological functions, with arrows indicating their influence on final phenotypic outcomes.

The genetic architecture underlying meat production traits in sheep represents a sophisticated biological system wherein multiple interconnected pathways coordinate growth, muscle development, fat deposition, and skeletal formation. Brief information about the genes documented in this review and their related pathways is provided in Supplementary Files 1, 2. This complex network involves numerous candidate genes that have been consistently reported across diverse sheep populations (Figure 2; Tables 1–3) and breeding programs worldwide, each contributing specific functional roles while participating in broader regulatory circuits that determine economically valuable traits.

Central to this genetic framework, myogenesis pathways control the fundamental processes of muscle development and ultimately determine muscle mass and composition that defines meat yield. The *MSTN* gene operates as a negative regulator within the transforming growth factor-beta signaling network, where its expression limits muscle growth through inhibition of satellite cell activation and myoblast proliferation. Consequently, when MSTN signaling is reduced through genetic variants, normal growth constraints are released, resulting in increased muscle fiber number and size, which translates directly to enhanced muscle mass and improved carcass composition. Furthermore, muscle-specific transcription factors *MEF2B, MYOD1,* and *MYF5* coordinate myogenic differentiation programs, controlling the expression of muscle-specific genes that determine fiber type characteristics and contractile properties. These regulatory networks interact synergistically with calcium-dependent signaling pathways involving troponin components such as *TNNC2* and myosin light chains including *MYL2,* which collectively determine muscle fiber contractility and ultimately influence meat texture and quality attributes. Complementing the myostatin pathway, the growth hormone regulatory network represents another critical system controlling overall growth performance and carcass development. This integrated pathway encompasses insulin-like growth factor 1 and its receptor, along with growth hormone and its corresponding receptor, functioning as a master regulator of somatic growth and metabolic processes through a sophisticated feedback system that regulates traits ranging from birth weight to final carcass characteristics. The signaling mechanism initiates with growth hormone binding to its receptor, triggering downstream activation of IGF1 synthesis in the liver and peripheral tissues. Subsequently, *IGF1* binds to its receptor, initiating intracellular signaling cascades that promote protein synthesis, muscle fiber development, and overall growth performance. This pathway directly influences carcass weight and yield by regulating cell proliferation, differentiation, and metabolism throughout the animal’s development, demonstrating dual influence on both muscle development and fat metabolism through intricate feedback mechanisms that ensure balanced growth processes responsive to physiological demands. In parallel, lipid metabolism pathways represent equally critical regulatory systems determining fat deposition patterns and meat quality characteristics. The triglyceride synthesis pathway, culminating in *DGAT1* and *DGAT2* enzymatic activity, controls the final steps of fat formation and storage. Notably, the diacylglycerol O-acyltransferase 1 gene catalyzes the final enzymatic step in triglyceride synthesis, demonstrating remarkable consistency in its associations with meat quality traits across sheep populations. Specific polymorphisms, particularly the K232A variant, have been extensively validated for their positive effects on loin meat yield and intramuscular fat content, directly influencing consumer-perceived meat quality. Concurrently, fatty acid-binding protein 4 plays a crucial role in fatty acid transport and cellular uptake, with genetic variations affecting both fat deposition patterns and meat quality characteristics. The fatty acid synthesis pathway, regulated by *FASN*, controls the production of fatty acids from acetyl-CoA precursors, while stearoyl-CoA desaturase introduces unsaturation into fatty acid chains, influencing membrane fluidity and meat quality attributes. Additionally, the bone morphogenetic protein family introduces an intriguing dimension to meat production genetics through its dual functionality in both skeletal development and adipogenesis. Specifically, *BMP2* and *BMP4* operate through specialized signaling pathways that simultaneously regulate fat tail development in certain sheep breeds while affecting bone formation and overall body size determination. This dual role becomes particularly relevant for breeds adapted to harsh environmental conditions, where fat reserves serve as critical survival mechanisms during periods of feed scarcity, thus representing an evolutionary adaptation that balances immediate production goals with long-term survival capacity. Moreover, skeletal development pathways contribute significantly to carcass characteristics through their control of bone formation and vertebral segmentation. The vertebral development genes *VRTN* and *NR6A1* regulate axial skeleton segmentation during embryogenesis, with genetic variants affecting the number of thoracic and lumbar vertebrae. Increased vertebral number directly correlates with longer carcass length and greater total carcass weight, providing measurable economic benefits. The *HOX* gene family provides positional information during development, ensuring proper spatial organization of skeletal structures that determine final body conformation and carcass geometry. Transcending individual pathway effects, master regulatory genes emerge as overarching controllers of multiple production traits through their influence on chromatin remodeling and transcriptional regulation. The *LCORL* and *NCAPG* genes appear consistently across genome-wide association studies investigating body size and growth traits, suggesting fundamental roles in determining mature body size and growth rate. These genes operate through epigenetic modifications and transcriptional control mechanisms, influencing the expression of numerous downstream targets involved in muscle development, bone growth, and overall body size determination. Similarly, *HMGA2* and *PLAG1* contribute additional layers of transcriptional control, particularly influencing growth-related gene expression patterns that determine mature body size and growth trajectory. The calpain-calpastatin proteolytic system represents a specialized post-mortem pathway that significantly influences meat quality and consumer acceptance. The calpain proteases, regulated by the calpastatin inhibitor encoded by the *CAST* gene, control protein degradation processes that occur after slaughter, determining the extent of myofibrillar protein breakdown that directly affects meat tenderness development during aging. Genetic variants affecting calpastatin expression influence the balance between protease activity and inhibition, ultimately determining the rate and extent of tenderization during post-mortem storage.

Furthermore, metabolic regulation pathways connect nutritional status with growth performance and carcass composition through genes such as adiponectin and leptin. These genes regulate energy homeostasis, fat distribution, appetite control, and energy expenditure, creating essential links between metabolic efficiency and production outcomes. The adiponectin pathway influences energy balance and fat distribution patterns, while leptin regulates appetite and energy expenditure, ensuring that growth processes remain aligned with nutritional resources and metabolic capacity.

**Figure 3:**
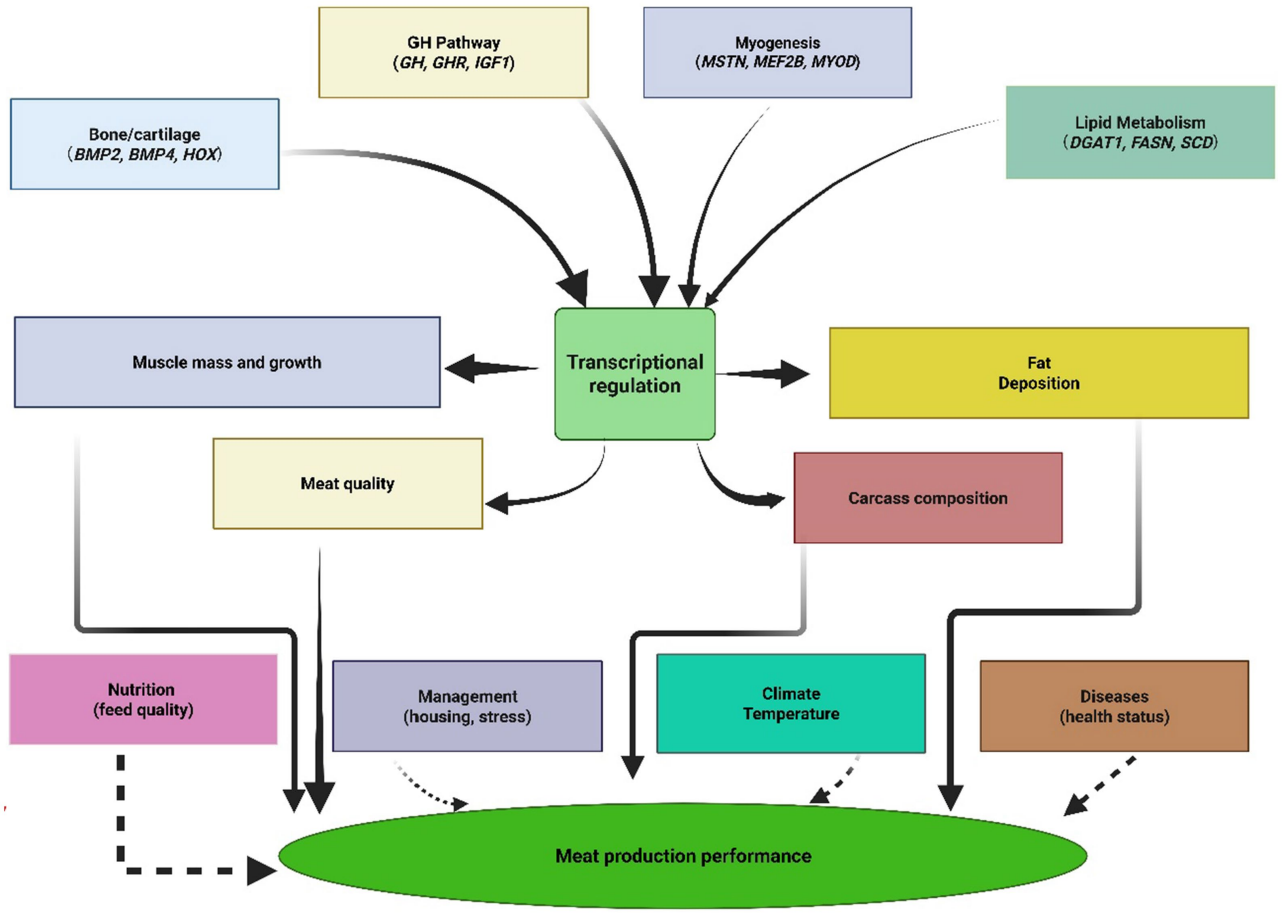
Biological pathway network showing how genetic factors interact with environmental influences to determine meat production traits. Solid arrows indicate direct genetic effects, while dashed lines show environmental modulation.

Environmental interactions add considerable complexity to these genetic systems, wherein genes like the fat mass and obesity-associated gene respond to nutritional status and environmental stressors, modulating their effects on growth and fat deposition based on external conditions. This environmental responsiveness indicates that gene expression can be influenced by factors including nutrition quality, health issues, temperature stress, and management practices, suggesting that optimal genetic selection programs must account for genotype-by-environment interactions to achieve consistent performance across diverse production systems (Figure 3).

The integration of these multiple pathways reveals that successful meat production genetics requires a systems-level approach rather than optimization of individual genes. Growth hormone signaling pathways interact extensively with muscle development regulators, while fat metabolism genes simultaneously influence meat quality characteristics and adaptive capacity. Bone morphogenetic proteins affect both skeletal development and fat deposition patterns, demonstrating the interconnected nature of physiological systems underlying meat production traits. Consequently, modern genomic selection approaches increasingly recognize these pathway interactions, moving beyond single-gene effects toward polygenic selection strategies that capture cumulative effects across multiple biological systems. This systems-level understanding provides the foundation for developing comprehensive genetic evaluation programs that can enhance meat production efficiency while maintaining genetic diversity and adaptive capacity essential for sustainable sheep production worldwide.

Based on published data, we concluded that current genetic research faces several significant limitations. Primarily, most genetic associations are discovered within specific breeds but lack validation across diverse populations. This creates limited applicability due to varying genetic backgrounds, distinct linkage disequilibrium patterns, and divergent population histories that cause population stratification effects. Furthermore, the field suffers from insufficient attention to epigenetic factors. DNA methylation patterns are largely ignored despite their significant influence on gene expression. Similarly, environmental interactions remain poorly understood, particularly how nutrition, climate, and management practices interact with genetic variants through complex epigenetic mechanisms. Moreover, the inheritance and influence of epigenetic marks across generations through transgenerational effects remains inadequately investigated. Consequently, the lack of comprehensive epigenome mapping across relevant tissues such as muscle, fat, and liver creates substantial knowledge gaps. This subsequently limits our understanding of tissue-specific regulatory mechanisms. Another critical limitation involves functional validation, where many identified single nucleotide polymorphisms may merely be in linkage disequilibrium with actual causal variants rather than being functionally relevant themselves. Additionally, insufficient experimental validation of how genetic variants actually affect gene function and protein activity perpetuates an oversimplified understanding of gene interactions within complex biological pathways.

To address these multifaceted challenges, future research must embrace multi-omics integration approaches. This includes combining epigenomics data such as DNA methylation, histone modifications, and chromatin accessibility with transcriptomics through expression quantitative trait loci mapping. Furthermore, incorporating proteomics and metabolomics will effectively link genetic variants to protein abundance and metabolite levels. Finally, investigating host-microbiome interactions that significantly affect production traits represents a critical research priority for advancing the field.

## 5 Conclusion and future research directions

This review has cataloged an extensive array of potential genes associated with meat production traits in sheep breeds globally. The identified genes—particularly those affecting vertebral development, muscle growth, and fat deposition—provide valuable targets for marker-assisted selection strategies to enhance sheep meat production efficiency. Future research should focus on validating these genetic associations across diverse populations and production environments to ensure broader applicability. Integration of advanced genomic technologies, including whole-genome sequencing and multi-omics approaches, will be crucial to understand the functional mechanisms underlying these genetic markers. Additionally, research examining gene–environment interactions and the role of epigenetic modifications on meat production traits deserves attention. Development of cost-effective genotyping platforms suitable for implementing these findings in resource-limited settings would further extend their practical value. Finally, the integration of consumer preferences with genetic selection represents a critical pathway for sustainable sheep breeding programs, where market demands increasingly favor specific meat quality attributes that should directly inform trait selection priorities. Consumer preference for leaner cuts drives selection for enhanced muscle development while reducing excessive fat deposition, while market premiums for higher carcass yield support prioritizing traits that increase carcass length and overall meat yield through improved skeletal development. Premium markets increasingly value optimal marbling for tenderness and flavor, requiring breeding programs to focus not just on fat deposition, but on achieving consumer-preferred intramuscular fat distribution that enhances both meat yield and quality characteristics. Growing consumer awareness of health benefits drives demand for favorable omega-3 to omega-6 fatty acid ratios, necessitating selection for optimized fatty acid synthesis and desaturation pathways to improve nutritional profiles, while market differentiation through functional meat products requires targeted selection of lipid metabolism traits. Post-mortem tenderization processes directly affect meat tenderness, a primary consumer concern, requiring selection programs to balance rapid growth with meat quality attributes that determine consumer satisfaction and repeat purchases. Future strategies should develop market-responsive breeding indices that weight genetic markers based on current consumer preferences and price premiums, establish feedback loops between consumer testing, market analysis, and breeding decisions, and consider regional market variations in trait preferences when implementing marker-assisted selection programs, ensuring that genetic improvements translate into economic value throughout the supply chain while meeting evolving consumer expectations for meat quality, nutrition, and eating experience.
