# Supplementary material for: Potential candidate genes influencing meat production phenotypic traits in sheep: a review
Source: Front Vet Sci. 2025 Jul 16;12:1616533. doi: 10.3389/fvets.2025.1616533 (PMC12307202; doi:10.3389/fvets.2025.1616533)
Supplement: Supplementary file 1 [file Data_Sheet_1.pdf]

Symbol	Ensembl G	Chr	Position (N	Descriptio	genomeSp	cds_length	transcript_	nExons
GPC1	ENSOARG	1	1.04083	glypican 1	6983	1473	1473	10
LRRFIP1	ENSOARG	1	3.178973	LRR bindir	91834	1698	2018	11
GIGYF2	ENSOARG	1	7.891667	GRB10 inte	129885	3915	4065	30
AKIRIN1	ENSOARG	1	13.48437	akirin 1 [Sc	8326	606	606	8
MACF1	ENSOARG	1	13.65827	microtubu	235505	21915	21915	101
SCMH1	ENSOARG	1	15.32305	Scm polyc	216305	1965	2232	15
CPT2	ENSOARG	1	27.46313	carnitine p	22431	1977	2096	5
GLIS1	ENSOARG	1	27.76836	GLIS family	97723	2187	2187	12
PRKAA2	ENSOARG	1	30.97709	protein kir	37445	1626	6069	8
JAK1	ENSOARG	1	40.0235	Janus kina	55707	3486	3796	24
LEPR	ENSOARG	1	40.76026	leptin rece	98057	3093	3154	19
LRRC8B	ENSOARG	1	66.17979	leucine ricl	21088	2415	2600	4
SLC16A1	ENSOARG	1	89.44885	solute carr	11172	1506	1523	4
OLFML3	ENSOARG	1	90.44327	olfactome	2816	1221	1577	3
CASQ2	ENSOARG	1	92.17199	calsequest	71123	1296	1861	11
PTGFRN	ENSOARG	1	93.47853	prostaglar	43996	2649	2649	9
TBX15	ENSOARG	1	95.56095	T-box trar	122901	1809	1809	8
CTSK	ENSOARG	1	99.66772	cathepsin	9692	993	994	7
HSPA6	ENSOARG	1	111.015	heat shock	1740	1740	1740	1
RBM11	ENSOARG	1	141.3597	RNA bindi	16588	741	1586	5
CADM2	ENSOARG	1	152.3475	cell adhesi	387062	1314	1314	10
SLC9C1	ENSOARG	1	175.311	solute carr	117375	3567	5102	30
ARHGAP3	ENSOARG	1	182.3928	Rho GTPas	117972	4353	4353	12
FSTL1	ENSOARG	1	183.4303	follistatin l	19064	936	2291	9
PARP14	ENSOARG	1	185.2752	poly(ADP-	50425	5388	7638	17
FGF12	ENSOARG	1	193.1428	fibroblast	413166	624	624	5
MASP1	ENSOARG	1	198.2368	mannan b	63665	1922	2115	14
ADIPOQ	ENSOARG	1	198.6187	Ovis aries	12545	852	2078	3
AHSG	ENSOARG	1	198.8062	alpha 2-H	7044	1272	1451	9
NAALADL	ENSOARG	1	209.3969	inactive N-	891235	2337	2345	13
PIK3CB	ENSOARG	1	248.4323	phosphati	125543	3225	3347	23
FAIM	ENSOARG	1	248.5725	Fas apopt	11289	744	744	5
MRAS	ENSOARG	1	248.7872	muscle RA	36488	630	648	5
RAB6B	ENSOARG	1	253.5715	RAB6B, me	10398	483	483	6
TF	ENSOARG	1	253.6337	serotransfe	39199	2115	2115	17
TNC	ENSOARG	2	8.640017	tenascin C	71187	7149	7286	30
TNFSF8	ENSOARG	2	8.805278	TNF super	26332	705	705	4
PTPN3	ENSOARG	2	13.79062	protein tyr	107652	2769	3038	26
SLC44A1	ENSOARG	2	17.57293	solute carr	108214	1912	1938	15
HSD17B3	ENSOARG	2	29.73553	hydroxyste	50357	933	933	12
PTCH1	ENSOARG	2	30.61957	patched 1	65486	4191	4191	24
GNRH1	ENSOARG	2	40.00037	gonadotrc	2757	291	291	3
LPL	ENSOARG	2	45.65212	lipoprotei	23785	1437	1437	10
TMOD1	ENSOARG	2	49.73294	tropomod	67041	1107	1107	9
HINT2	ENSOARG	2	52.42111	histidine tr	2279	492	492	5
SPAG8	ENSOARG	2	52.4233	sperm ass	3178	1306	1794	6
NPR2	ENSOARG	2	52.42384	natriuretic	21334	3201	7142	22
TPM2	ENSOARG	2	52.53746	tropomyos	7494	855	1090	9
TLE4	ENSOARG	2	56.63734	TLE family	159020	2139	2530	19
PGM5	ENSOARG	2	67.71701	phosphog	204683	1704	2699	11
DOCK8	ENSOARG	2	68.03263	dedicator	264338	6200	6206	47
DMRT1	ENSOARG	2	68.63081	doublesex	53520	996	996	7
RFX3	ENSOARG	2	71.00315	regulatory	321607	2376	3101	19
GLIS3	ENSOARG	2	71.59265	GLIS family	470673	2799	2898	10
JAK2	ENSOARG	2	72.78181	Janus kina	113513	3402	3507	24
KDM4C	ENSOARG	2	74.1725	lysine dem	363630	3162	3179	21
FREM1	ENSOARG	2	82.797	FRAS1 rela	150904	6564	6564	36

ZNF395	ENSOARG	2	101.6123	zinc finger	15585	1530	1530	10
KIF13B	ENSOARG	2	102.4107	kinesin fan	122855	5040	5040	38
GDF-8	ENSOARG	2	118.1444	myostatin	4991	1128	1128	3
ORMDL1	ENSOARG	2	118.4349	ORMDL sp	8326	462	469	3
RBM45	ENSOARG	2	130.8771	RNA bindi	22180	1416	6669	10
CERS6	ENSOARG	2	139.4866	ceramide s	344892	1125	1127	12
FIGN	ENSOARG	2	144.8288	fidgetin, m	127525	2280	2695	2
IFIH1	ENSOARG	2	146.3432	interferon	61235	3070	3070	16
ZEB2	ENSOARG	2	164.4408	zinc finger	41755	3582	3582	13
KYNU	ENSOARG	2	165.9433	kynurenina	112039	1239	1245	12
ANKRD44	ENSOARG	2	198.2419	ankyrin rep	185688	3015	3208	27
PLCL1	ENSOARG	2	199.1737	phospholip	194200	2967	2967	6
ALS2	ENSOARG	2	202.9259	alsin Rho g	48335	4974	5932	33
CREB1	ENSOARG	2	208.5639	cAMP resp	49778	1005	1537	8
FN1	ENSOARG	2	216.3094	fibronectir	68784	7437	7438	46
MREG	ENSOARG	2	216.944	melanoreg	68716	645	645	5
DIS3L2	ENSOARG	2	232.7492	DIS3 like 3	298916	2649	4535	20
AK2	ENSOARG	2	233.7309	adenylate	22109	684	754	7
FNDC5	ENSOARG	2	233.8894	fibronectir	6593	618	834	6
FABP3	ENSOARG	2	235.1355	fatty acid b	10469	402	1381	6
TMEM50A	ENSOARG	2	240.5465	transmemb	15426	474	1160	7
MYOM3	ENSOARG	2	241.6913	myomesin	48157	4320	4338	37
RAP1GAP	ENSOARG	2	244.025	RAP1 GTPa	30419	2187	2188	22
NSMF	ENSOARG	3	0.525717	NMDA rec	8518	1176	1176	8
ANGPTL2	ENSOARG	3	8.961593	angiopoiet	21540	1398	1398	5
NR6A1	ENSOARG	3	11.2386	nuclear rec	32960	1305	1305	8
ROCK2	ENSOARG	3	20.05871	Rho associ	83600	4068	4570	33
LPIN1	ENSOARG	3	20.54739	lipin 1 [Sou	47213	2814	2817	22
POMC	ENSOARG	3	32.46666	proopiome	3287	765	785	3
FOSL2	ENSOARG	3	35.22171	FOS like 2,	18732	984	1370	4
GFPT1	ENSOARG	3	38.82999	glutamine-	53157	1812	4822	18
PROKR1	ENSOARG	3	39.58001	prokinetici	12196	1284	1342	2
ETAA1	ENSOARG	3	40.76892	ETAA1 act	13519	2712	2712	6
MEIS1	ENSOARG	3	41.64887	Meis home	151021	1286	6878	12
POLR1B	ENSOARG	3	60.29851	RNA polyr	20541	3432	3432	15
ST6GAL2	ENSOARG	3	61.03193	ST6 beta-g	24332	1518	1518	16
ASB3	ENSOARG	3	70.17942	ankyrin rep	98050	1584	1717	10
THADA	ENSOARG	3	80.57517	THADA arr	327833	5841	9867	38
SPAST	ENSOARG	3	91.66787	spastin [So	50986	1875	1875	18
TGFA	ENSOARG	3	92.43132	transformi	108270	483	717	6
MOB1A	ENSOARG	3	95.9183	MOB kinas	21793	693	693	6
MGAT4A	ENSOARG	3	102.4191	alpha-1,3-	112642	1608	1608	15
BCL2L11	ENSOARG	3	105.2056	BCL2 like 1	45607	588	1414	4
TPH2	ENSOARG	3	107.854	tryptophar	99194	1683	1683	11
TRHDE	ENSOARG	3	108.2356	thyrotropir	449387	2616	2739	19
MYF5	ENSOARG	3	116.6203	myogenic	2123	768	876	3
KITLG	ENSOARG	3	124.6696	KIT ligand	92839	825	862	9
BTG1	ENSOARG	3	128.3821	BTG anti-p	369	369	369	1
SOCS2	ENSOARG	3	129.7205	suppressor	1993	594	594	2
CRADD	ENSOARG	3	129.8203	CASP2 and	188784	600	606	2
IGFBP6	ENSOARG	3	133.2133	insulin like	3587	717	717	6
ACVRL1	ENSOARG	3	134.2294	activin A re	6770	1161	1161	7
KMT2D	ENSOARG	3	136.9829	lysine meth	36151	16053	16450	66
ADCY6	ENSOARG	3	137.2063	adenylate	18241	3501	3647	22
TMEM117	ENSOARG	3	142.0845	transmemb	596537	1174	1409	6
CNTN1	ENSOARG	3	145.8274	contactin 1	183560	3068	3332	23
LRRK2	ENSOARG	3	146.5516	leucine ricl	213005	7608	8609	51
LLPH	ENSOARG	3	153.5447	LLP homol	3564	387	387	2

MSRB3	ENSOARG	3	154.2192	methionin	178753	555	692	7
SLC16A7	ENSOARG	3	159.5339	solute carr	132886	1446	1574	5
LRIG3	ENSOARG	3	160.4073	leucine ricl	51398	3378	3378	20
IGF1	ENSOARG	3	171.2684	insulin like	59353	516	516	5
PDE3A	ENSOARG	3	193.922	phosphod	306029	2490	2490	16
EPS8	ENSOARG	3	199.0257	epidermal	125884	2538	2742	21
CACNA1C	ENSOARG	3	212.4273	calcium vo	365614	6444	6786	54
SHISAL1	ENSOARG	3	218.961	shisa like 1	13950	627	627	7
VSTM2A	ENSOARG	4	0.436303	V-set and	22882	723	902	5
DDC	ENSOARG	4	5.326272	dopa deca	84759	1546	3937	14
PDK4	ENSOARG	4	12.76876	pyruvate c	12689	1236	2627	11
COL28A1	ENSOARG	4	15.31763	collagen ty	179151	3459	3496	34
DGKB	ENSOARG	4	22.45196	diacylglyce	806561	2088	2182	22
PRPS1L1	ENSOARG	4	26.60702	phosphori	990	990	990	1
CACNA2D	ENSOARG	4	38.13075	calcium vo	535278	3216	3457	41
SFRP4	ENSOARG	4	49.72351	secreted fr	9473	1041	1041	6
CTTNBP2	ENSOARG	4	50.43254	cortactin b	171399	4782	4947	25
ST7	ENSOARG	4	51.10867	suppressic	159512	1734	1734	15
IFRD1	ENSOARG	4	55.89156	interferon	29505	1353	6260	13
GHRHR	ENSOARG	4	65.35035	growth ho	14460	1278	1278	14
HOXA10	ENSOARG	4	68.85804	homeobox	5453	1242	4252	2
HOXA9	ENSOARG	4	68.86779	homeobox	1876	819	819	2
CAMK2B	ENSOARG	4	77.06821	calcium/ca	90257	1995	1995	26
WNT16	ENSOARG	4	85.86273	Wnt family	9699	1074	1074	5
FAM3C	ENSOARG	4	85.88744	FAM3 met	36405	684	1683	9
LEP	ENSOARG	4	92.50829	leptin [Sou	13894	615	615	3
PLXNA4	ENSOARG	4	95.78831	plexin A4 [	415744	5652	7494	37
CNOT6	ENSOARG	5	0.34343	CCR4-NOT	65201	1629	1680	11
MEF2B	ENSOARG	5	3.860938		3388	972	972	6
INSR	ENSOARG	5	13.7336	insulin rec	145550	4106	5230	21
RETN	ENSOARG	5	14.18529	Ovis aries	980	330	330	3
VAV1	ENSOARG	5	15.32152	vav guanir	64478	2526	2526	27
SLC22A4	ENSOARG	5	19.64046	solute carr	41756	1737	1737	10
PROP1	ENSOARG	5	37.29857	PROP pair	5714	681	3610	3
SAR1B	ENSOARG	5	43.50004	secretion a	25019	597	1323	7
LECT2	ENSOARG	5	44.74919	leukocyte	11943	456	456	5
TRPC7	ENSOARG	5	45.01306	transient r	140361	2589	2589	11
HTR4	ENSOARG	5	57.37907	5-hydroxy	204612	1206	1283	7
PIGY	ENSOARG	5	58.45172	phosphatic	213	213	213	1
PPARGC1	ENSOARG	5	58.67006	PPARG coa	28940	3051	3051	15
CSF1R	ENSOARG	5	58.89683	colony stir	30275	2889	2889	21
PDGFRB	ENSOARG	5	58.9819	platelet de	35472	3312	3435	23
CDX1	ENSOARG	5	59.02727	caudal typ	21649	798	6141	3
SYNPO	ENSOARG	5	59.50133	synaptopo	32728	2622	2622	5
SPARC	ENSOARG	5	60.40521	secreted p	22402	1389	2259	10
MCTP1	ENSOARG	5	91.37849	multiple C	370517	2328	2328	23
CAST	ENSOARG	5	93.3544	calpastatir	129689	2361	2701	32
CAMK4	ENSOARG	5	107.5409	calcium/ca	271709	1002	1004	10
PITX2	ENSOARG	6	14.93443	paired like	21565	966	3785	4
ELOVL6	ENSOARG	6	15.30471	ELOVL fatt	134475	795	814	4
CFI	ENSOARG	6	15.7088	compleme	44215	1840	1840	15
GSTCD	ENSOARG	6	19.46442	glutathion	128784	1896	1896	11
BMPR1B	ENSOARG	6	29.36195	bone morp	86133	1677	3480	12
CCSER1	ENSOARG	6	33.6622	coiled-coil	833073	2232	2782	10
GPRIN3	ENSOARG	6	35.51129	GPRIN fam	2343	2343	2343	1
PIGY	ENSOARG	6	36.19302	PIGY upstr	213	213	213	1
SPP1	ENSOARG	6	36.65173	secreted p	6555	840	988	7
LAP3	ENSOARG	6	37.09232	leucine am	26256	1569	4346	13

MED28	ENSOARG	6	37.1264	mediator c	5869	537	537	4
FAM184B	ENSOARG	6	37.13867	family with	119090	3795	3795	24
NCAPG	ENSOARG	6	37.25655	non-SMC	77304	3093	3093	21
DCAF16	ENSOARG	6	37.27864	DDB1 and	651	651	651	1
LCORL	ENSOARG	6	37.36524	ligand dep	87097	1683	1683	7
KCNIP4	ENSOARG	6	40.15663	potassium	242840	804	804	9
PPARGC1	ENSOARG	6	43.22682	PPARG coa	104535	2460	2460	14
RBPJ	ENSOARG	6	45.62224	recombina	108932	1464	1464	11
ARAP2	ENSOARG	6	55.46014	ArfGAP wi	196424	5127	6893	33
DTHD1	ENSOARG	6	55.69717	death dom	86949	2760	2760	10
GRXCR1	ENSOARG	6	61.64365	glutaredox	136982	873	873	4
PDGFRA	ENSOARG	6	69.7358	platelet de	36960	3272	4114	22
ADGRL3	ENSOARG	6	77.25253	adhesion G	314427	3996	3996	22
FGF5	ENSOARG	6	94.5844	fibroblast g	21176	600	600	4
SCD5	ENSOARG	6	97.07807	stearoyl-C	69659	780	780	4
EVC2	ENSOARG	6	103.1302	EvC ciliary	159136	3783	3783	24
ITGA11	ENSOARG	7	14.89898	integrin su	134377	3554	3570	30
NDRG2	ENSOARG	7	23.30759	NDRG fam	6326	1095	1282	16
RYR3	ENSOARG	7	25.72018	ryanodine	471754	14607	14607	103
ZNF770	ENSOARG	7	27.43946	zinc finger	2076	2076	2076	1
CAPN3	ENSOARG	7	34.74715	calpain 3 [	58058	2469	6048	24
DNAAF2	ENSOARG	7	39.7544	dynein axo	6368	2283	2283	5
NEDD4	ENSOARG	7	51.43681	NEDD4 E3	125937	2553	2663	28
SLC27A2	ENSOARG	7	57.02226	solute carr	51935	1857	2387	10
SHC4	ENSOARG	7	58.24647	SHC adapt	128923	1893	1893	12
BMP4	ENSOARG	7	63.45034	bone morp	5883	1230	3863	3
SIX1	ENSOARG	7	69.73797	SIX homeo	3116	1068	1068	2
KCNH5	ENSOARG	7	71.87345	potassium	380989	2967	2967	11
SYNE2	ENSOARG	7	73.00174	spectrin re	313063	19578	20480	110
SPTB	ENSOARG	7	73.80114	spectrin be	68997	6987	6987	38
ZFP36L1	ENSOARG	7	77.52219	ZFP36 ring	3453	888	888	4
ACTN1	ENSOARG	7	77.57778	actinin alp	41690	2661	2661	25
ABCD4	ENSOARG	7	82.53986	ATP bindir	14174	1821	1821	19
SYNDIG1L	ENSOARG	7	82.62226	synapse di	5487	717	3795	3
LTBP2	ENSOARG	7	82.70507	latent tran	107563	5886	5886	37
TGFB3	ENSOARG	7	84.19463	transformi	23204	1359	1711	7
TMEM63C	ENSOARG	7	85.35783	transmeml	58920	2412	2568	23
TSHR	ENSOARG	7	89.25842	thyroid stir	173454	2295	2603	10
COL12A1	ENSOARG	8	1.763844	collagen ty	117345	9378	11019	66
PHIP	ENSOARG	8	5.690526	pleckstrin	110071	5124	7396	36
UBE3D	ENSOARG	8	9.947372	ubiquitin p	165210	990	2012	10
ME1	ENSOARG	8	10.27689	malic enzy	184355	1638	1638	13
FABP7	ENSOARG	8	15.54071	fatty acid b	3493	399	399	4
FBXL4	ENSOARG	8	37.90055	F-box and	74036	1866	2489	9
MAP3K5	ENSOARG	8	61.57786	mitogen-a	229228	3675	5514	30
TBXT	ENSOARG	8	87.79614	T-box tran	9410	1335	1335	9
TRAPPC9	ENSOARG	9	16.04998	trafficking	319237	3354	7615	21
KHDRBS3	ENSOARG	9	19.35855	KH RNA bi	98061	1035	1035	10
PLAG1	ENSOARG	9	36.16509	PLAG1 zin	44634	1506	1909	4
CHCHD7	ENSOARG	9	36.21331	coiled-coil	2793	276	276	3
CLVS1	ENSOARG	9	39.65706	clavesin 1	153262	1065	1238	5
NKAIN3	ENSOARG	9	40.54556	sodium/po	256285	615	2870	6
CYP7B1	ENSOARG	9	42.11801	25-hydrox	173028	1521	5455	6
ZNF704	ENSOARG	9	56.69832	zinc finger	189395	1167	1269	8
FABP4	ENSOARG	9	57.53653	fatty acid b	4518	399	399	4
FZD6	ENSOARG	9	73.80195	frizzled cla	192120	2172	2172	7
CPQ	ENSOARG	9	79.58331	carboxype	439100	1452	1988	7
SLC25A30	ENSOARG	10	15.91056	solute carr	13868	876	1014	10

UBL3	ENSOARG	10	31.05227	ubiquitin li	43400	363	644	5
ATP8A2	ENSOARG	10	33.60797	phospholi	397897	3141	3531	36
FGF9	ENSOARG	10	35.58384	fibroblast	25573	644	692	5
NALCN	ENSOARG	10	76.92294	sodium lea	279882	5217	5217	43
METTL21(	ENSOARG	10	78.27473	methyltrar	8524	774	774	4
ACACA	ENSOARG	11	13.02944	acetyl-CoA	228431	6774	7019	53
SLC6A4	ENSOARG	11	20.91339	solute carr	20052	1893	2004	13
KIF1C	ENSOARG	11	26.00456	kinesin fan	21323	3318	5588	21
CHRNB1	ENSOARG	11	26.75357	cholinergic	8213	1527	1527	11
COL1A1	ENSOARG	11	35.99383	collagen ty	16206	4425	4425	49
IGF2BP1	ENSOARG	11	37.0589	insulin like	40846	1734	3142	15
HOXB13	ENSOARG	11	37.33723	homeobox	1758	858	858	2
KRT20	ENSOARG	11	40.63778	keratin 20	8974	1290	1290	8
KRT23	ENSOARG	11	40.69064	keratin 23	16595	1248	1248	8
ACLY	ENSOARG	11	41.53771	ATP citrate	37290	3306	3306	29
STAT3	ENSOARG	11	41.90305	signal tran	31789	2478	4097	23
MAP3K14	ENSOARG	11	44.33798	mitogen-a	18991	2862	2882	15
GH	ENSOARG	11	47.54017	growth ho	1631	654	654	5
BAIAP2	ENSOARG	11	50.72832	BAR/IMD (	40820	1602	1602	15
FBF1	ENSOARG	11	54.7079	Fas bindin	19343	3546	3546	32
MYOG	ENSOARG	12	0.196023	myogenin	1937	585	585	4
TGFB2	ENSOARG	12	19.93487	transformi	90876	1248	1795	7
AKT3	ENSOARG	12	31.52009	AKT serine	263864	1440	1686	13
PER3	ENSOARG	12	43.65505	period circ	68163	3480	4298	21
CAMTA1	ENSOARG	12	43.73779	calmodulin	126314	4467	9205	17
AADACL3	ENSOARG	12	52.79758	arylacetam	10551	1245	3093	4
PAPPA2	ENSOARG	12	55.75632	pappalysin	314396	5382	6322	22
ASTN1	ENSOARG	12	56.09406	astrotactin	236581	3657	4921	22
KCNH1	ENSOARG	12	70.25272	potassium	501333	3042	3198	12
PLCB1	ENSOARG	13	0.666266	phospholi	488262	3354	3419	31
PLXDC2	ENSOARG	13	20.37218	plexin don	439565	1590	5435	14
SYNDIG1	ENSOARG	13	41.53564	synapse di	111223	705	4588	4
prp	ENSOARG	13	46.22525	prion prot	771	771	771	1
CDS2	ENSOARG	13	46.62544	CDP-diacy	42303	1338	1338	13
BMP2	ENSOARG	13	48.46223	bone mor	10368	1278	1278	2
CPXM1	ENSOARG	13	51.7078	probable c	6453	2184	2427	14
TMC2	ENSOARG	13	51.87596	transmeml	100471	2670	2715	21
REM1	ENSOARG	13	60.37753	RRAD and	8840	1050	1050	5
ACSS2	ENSOARG	13	63.748	acyl-CoA s	43525	2126	2126	18
GHRH	ENSOARG	13	65.77181	growth ho	4613	327	327	4
TNNC2	ENSOARG	13	74.13646	troponin C	1052	447	447	4
EYA2	ENSOARG	13	75.06677	EYA transc	261691	1614	2111	16
SULF2	ENSOARG	13	75.72673	sulfatase 2	44395	2184	6424	18
NFATC2	ENSOARG	13	78.81542	nuclear fac	77654	2439	2439	14
MYLK3	ENSOARG	14	14.55096	myosin lig	43871	2349	2349	13
FTO	ENSOARG	14	21.52499	FTO alpha	429005	1518	3656	9
GOT2	ENSOARG	14	25.86855	glutamic-c	21948	1293	2126	10
TGFB1	ENSOARG	14	49.65975	transformi	14979	1144	1506	8
LIPE	ENSOARG	14	49.93022	lipase E, hc	19211	2541	2633	10
EPN1	ENSOARG	14	59.13169	epsin 1 [Sc	7348	1302	1304	10
PDGFD	ENSOARG	15	3.848546	platelet de	285453	1113	5636	7
MMP13	ENSOARG	15	5.258462	matrix met	12419	1443	2450	10
MMP12	ENSOARG	15	5.352875	matrix met	9353	1413	1807	10
CRYAB	ENSOARG	15	21.67487	crystallin a	2882	528	528	3
APOA5	ENSOARG	15	26.93007	apolipopro	1775	1074	1074	7
MYOD1	ENSOARG	15	34.37053	myogenic	595	594	594	2
PDE2A	ENSOARG	15	50.33907	phosphod	33610	2817	2817	31
ARAP1	ENSOARG	15	50.44262	ArfGAP wi	40887	4299	4871	41

UCP3	ENSOARG	15	51.62279	uncouplin	5932	936	1030	6
PGM2L1	ENSOARG	15	51.88535	phosphog	54869	1890	2099	14
SLCO2B1	ENSOARG	15	52.57257	solute carr	36762	2187	2187	14
DGAT2	ENSOARG	15	53.33791	diacylglyce	18234	1170	1170	9
WT1	ENSOARG	15	61.32649	WT1 trans	48532	1353	1353	13
ALX4	ENSOARG	15	72.55606	ALX home	50196	915	915	6
FGF18	ENSOARG	16	3.266357	fibroblast	34540	645	688	4
PIK3R1	ENSOARG	16	11.38514	phosphoir	85529	2178	7239	16
FST	ENSOARG	16	25.63086	follistatin [	5265	1035	1129	6
MRPS30	ENSOARG	16	30.0423	mitochonc	9224	1308	2341	7
GHR	ENSOARG	16	31.83293	growth ho	167513	1956	1956	10
PRKAA1	ENSOARG	16	33.6176	protein kir	25517	1702	1702	10
CDH10	ENSOARG	16	48.7494	cadherin-1	113354	2367	2492	11
CDH18	ENSOARG	16	53.23095	cadherin 1	622035	2121	7384	14
UCP1	ENSOARG	17	16.84768	uncouplin	5996	918	918	6
SETD7	ENSOARG	17	17.83113	SET domai	46797	1101	1194	8
MYL2	ENSOARG	17	54.29557	myosin lig	7501	501	594	7
RAB21	ENSOARG	17	59.80242	RAB21, me	666	666	666	1
CFAP73	ENSOARG	17	60.82349	cilia and fl	9484	909	909	8
THOC5	ENSOARG	17	68.23603	THO comp	27877	1978	2128	20
LRRK1	ENSOARG	18	4.54634	leucine ricl	134654	6003	6236	36
IGF1R	ENSOARG	18	7.159507	insulin like	60238	3366	3366	25
ACAN	ENSOARG	18	19.52082	aggrecan [	39201	6621	6621	19
PLIN1	ENSOARG	18	20.19284	perilipin 1	11488	1554	1568	9
SH3GL3	ENSOARG	18	22.98607	SH3 doma	53822	1002	1002	8
RALGAPA	ENSOARG	18	44.67981	Ral GTPase	192617	6135	6158	40
SSTR1	ENSOARG	18	47.23451	somatosta	1173	1173	1173	1
MFAP1	ENSOARG	18	54.38406	microfibril	11208	1320	1320	9
SERPINA1	ENSOARG	18	58.04661	serpin fam	30290	1293	1293	5
BDKRB2	ENSOARG	18	59.81013	bradykinin	4578	1164	1164	2
GSKIP	ENSOARG	18	59.95467	GSK3B inte	4263	420	420	2
BCL11B	ENSOARG	18	62.84722	BAF chron	83745	1947	1947	10
SETD3	ENSOARG	18	63.07387	SET domai	75860	1767	2542	14
DLK1	ENSOARG	18	64.32821	delta like r	6712	762	762	5
TGFBR2	ENSOARG	19	5.059654	transformi	50420	1707	1707	6
ARPP21	ENSOARG	19	9.569024	cAMP regu	114294	2418	2456	18
TRAK1	ENSOARG	19	14.38639	trafficking	99042	2772	3071	17
LMCD1	ENSOARG	19	17.79856	LIM and cy	30446	1083	2435	8
CHL1	ENSOARG	19	25.92374	cell adhesi	345220	3687	3884	28
CNTN3	ENSOARG	19	27.10712	contactin 3	294561	3087	3087	22
MAGI1	ENSOARG	19	35.6384	membrane	415091	4148	6175	24
ADAMTS9	ENSOARG	19	36.78383	ADAM me	158720	5748	5749	39
MST1	ENSOARG	19	50.45361	macropha	5794	2511	2511	21
KLF15	ENSOARG	19	59.76504	Kruppel lik	1076	975	975	2
HMGA1	ENSOARG	20	8.43895	high mobi	2623	288	288	4
PPARD	ENSOARG	20	9.408372	peroxisom	84139	1326	1512	8
SRF	ENSOARG	20	16.85457	serum resp	4271	1062	1062	6
TFAP2B	ENSOARG	20	23.15358	transcriptio	24609	1410	1438	8
ELOVL5	ENSOARG	20	25.19307	ELOVL fatt	24832	888	896	8
PRL	ENSOARG	20	34.25808	prolactin [	8821	696	1175	5
CAP2	ENSOARG	20	38.78631	cyclase ass	145290	1467	1836	13
DTNBP1	ENSOARG	20	40.34043	dystrobrev	90510	1053	1053	12
ELOVL2	ENSOARG	20	44.2161	ELOVL fatt	17880	885	885	7
BMP6	ENSOARG	20	46.48614	bone mor	26013	1023	1023	8
PAK1	ENSOARG	21	17.99407	p21 (RAC1	77100	1635	1656	14
LUZP2	ENSOARG	21	19.46635	leucine zip	275887	795	873	10
FADS3	ENSOARG	21	39.7981	fatty acid	14956	1326	1326	15
LGALS12	ENSOARG	21	41.1806	galectin 12	8592	984	984	9

FLRT1	ENSOARG	21	41.74703	fibronectir	1905	1779	1779	3
MAP3K11	ENSOARG	21	43.09007	mitogen-a	15175	2577	2577	11
PCDH15	ENSOARG	22	4.372641	protocadh	964569	5355	5544	38
SCD	ENSOARG	22	20.40924	stearoyl-C	12600	1101	2555	6
CPXM2	ENSOARG	22	42.38016	carboxype	137979	2208	2208	15
CYP2E1	ENSOARG	22	50.69231	cytochrom	14111	1497	3771	10
MOCOS	ENSOARG	23	21.16778	molybdenu	62659	2550	2550	17
ELP2	ENSOARG	23	21.24093	elongator	57726	3012	5577	25
NPC1	ENSOARG	23	33.34343	NPC intrac	44758	3828	3828	27
SETBP1	ENSOARG	23	44.57426	SET bindin	385367	3189	4081	4
MC4R	ENSOARG	23	59.38558	melanocor	999	999	999	1
SSTR5	ENSOARG	24	0.812728	somatosta	1139	1044	1044	4
IGFALS	ENSOARG	24	1.353748	insulin like	1976	1302	1302	3
ABAT	ENSOARG	24	7.601608	4-aminobu	36842	1503	1503	15
MYH11	ENSOARG	24	14.09495	myosin he	109261	5850	6085	40
HSPB1	ENSOARG	24	34.25625	heat shock	1472	555	555	3
MYL10	ENSOARG	24	35.08559	myosin lig	12031	531	3700	6
ACTA1	ENSOARG	25	0.773585	actin alpha	2479	1134	1243	7
GGPS1	ENSOARG	25	8.107696	geranylger	10170	903	1517	4
CTNNA3	ENSOARG	25	22.14808	catenin alp	986939	1683	1683	12
GDF2	ENSOARG	25	41.83578	growth dif	4061	1278	1278	2
GDF10	ENSOARG	25	41.84947	growth dif	11897	1437	1437	3
ACSL1	ENSOARG	26	13.92175	acyl-CoA s	42439	2205	2205	20
FAT1	ENSOARG	26	15.15625	FAT atypic	107055	13776	13776	29
FRG1	ENSOARG	26	17.86987	FSHD regi	12968	783	783	10
BAG4	ENSOARG	26	31.99657	BAG cocha	24189	1317	1317	6
KAT6A	ENSOARG	26	35.33396	lysine acet	101608	5808	5808	19
TOP2B	ENSOARG	26	38.64676	DNA topo	56235	4842	9441	37
FRMPD4	ENSOARG	X	10.04721	FERM and	205789	4515	4594	16
WAS	ENSOARG	X	53.13954	WASP acti	7302	1395	1725	13
OGT	ENSOARG	X	60.79157	O-linked N	38309	3144	3174	22
CAPN6	ENSOARG	X	117.4631	calpain 6 [	17018	1926	2169	12
COL4A6	ENSOARG	X	119.737	collagen ty	339590	5098	5258	46
NA	Not mapp	NA	NA	NA	NA	NA	NA	NA
NA	Not mapp	NA	NA	NA	NA	NA	NA	NA
NA	Not mapp	NA	NA	NA	NA	NA	NA	NA
NA	Not mapp	NA	NA	NA	NA	NA	NA	NA
NA	Not mapp	NA	NA	NA	NA	NA	NA	NA
NA	Not mapp	NA	NA	NA	NA	NA	NA	NA
NA	Not mapp	NA	NA	NA	NA	NA	NA	NA
NA	Not mapp	NA	NA	NA	NA	NA	NA	NA
NA	Not mapp	NA	NA	NA	NA	NA	NA	NA
NA	Not mapp	NA	NA	NA	NA	NA	NA	NA
NA	Not mapp	NA	NA	NA	NA	NA	NA	NA
NA	Not mapp	NA	NA	NA	NA	NA	NA	NA
NA	Not mapp	NA	NA	NA	NA	NA	NA	NA
NA	Not mapp	NA	NA	NA	NA	NA	NA	NA
NA	Not mapp	NA	NA	NA	NA	NA	NA	NA
NA	Not mapp	NA	NA	NA	NA	NA	NA	NA
NA	Not mapp	NA	NA	NA	NA	NA	NA	NA
NA	Not mapp	NA	NA	NA	NA	NA	NA	NA
NA	Not mapp	NA	NA	NA	NA	NA	NA	NA
NA	Not mapp	NA	NA	NA	NA	NA	NA	NA
NA	Not mapp	NA	NA	NA	NA	NA	NA	NA
NA	Not mapp	NA	NA	NA	NA	NA	NA	NA
NA	Not mapp	NA	NA	NA	NA	NA	NA	NA
NA	Not mapp	NA	NA	NA	NA	NA	NA	NA
NA	Not mapp	NA	NA	NA	NA	NA	NA	NA

NA	Not mapp	NA	NA	NA	NA	NA	NA	NA
NA	Not mapp	NA	NA	NA	NA	NA	NA	NA
NA	Not mapp	NA	NA	NA	NA	NA	NA	NA
NA	Not mapp	NA	NA	NA	NA	NA	NA	NA
NA	Not mapp	NA	NA	NA	NA	NA	NA	NA
NA	Not mapp	NA	NA	NA	NA	NA	NA	NA
NA	Not mapp	NA	NA	NA	NA	NA	NA	NA
NA	Not mapp	NA	NA	NA	NA	NA	NA	NA
NA	Not mapp	NA	NA	NA	NA	NA	NA	NA
NA	Not mapp	NA	NA	NA	NA	NA	NA	NA
NA	Not mapp	NA	NA	NA	NA	NA	NA	NA
NA	Not mapp	NA	NA	NA	NA	NA	NA	NA
NA	Not mapp	NA	NA	NA	NA	NA	NA	NA
NA	Not mapp	NA	NA	NA	NA	NA	NA	NA
NA	Not mapp	NA	NA	NA	NA	NA	NA	NA
NA	Not mapp	NA	NA	NA	NA	NA	NA	NA
NA	Not mapp	NA	NA	NA	NA	NA	NA	NA
NA	Not mapp	NA	NA	NA	NA	NA	NA	NA
NA	Not mapp	NA	NA	NA	NA	NA	NA	NA
NA	Not mapp	NA	NA	NA	NA	NA	NA	NA
NA	Not mapp	NA	NA	NA	NA	NA	NA	NA
NA	Not mapp	NA	NA	NA	NA	NA	NA	NA
NA	Not mapp	NA	NA	NA	NA	NA	NA	NA
NA	Not mapp	NA	NA	NA	NA	NA	NA	NA
NA	Not mapp	NA	NA	NA	NA	NA	NA	NA
NA	Not mapp	NA	NA	NA	NA	NA	NA	NA
NA	Not mapp	NA	NA	NA	NA	NA	NA	NA
NA	Not mapp	NA	NA	NA	NA	NA	NA	NA
NA	Not mapp	NA	NA	NA	NA	NA	NA	NA
NA	Not mapp	NA	NA	NA	NA	NA	NA	NA
NA	Not mapp	NA	NA	NA	NA	NA	NA	NA
NA	Not mapp	NA	NA	NA	NA	NA	NA	NA
NA	Not mapp	NA	NA	NA	NA	NA	NA	NA
NA	Not mapp	NA	NA	NA	NA	NA	NA	NA
NA	Not mapp	NA	NA	NA	NA	NA	NA	NA
NA	Not mapp	NA	NA	NA	NA	NA	NA	NA
NA	Not mapp	NA	NA	NA	NA	NA	NA	NA
NA	Not mapp	NA	NA	NA	NA	NA	NA	NA
NA	Not mapp	NA	NA	NA	NA	NA	NA	NA
NA	Not mapp	NA	NA	NA	NA	NA	NA	NA
NA	Not mapp	NA	NA	NA	NA	NA	NA	NA
NA	Not mapp	NA	NA	NA	NA	NA	NA	NA
NA	Not mapp	NA	NA	NA	NA	NA	NA	NA
NA	Not mapp	NA	NA	NA	NA	NA	NA	NA
