# Supplementary material for: Potential candidate genes influencing meat production phenotypic traits in sheep: a review
Source: Front Vet Sci. 2025 Jul 16;12:1616533. doi: 10.3389/fvets.2025.1616533 (PMC12307202; doi:10.3389/fvets.2025.1616533)
Supplement: Supplementary file 2 [file Data_Sheet_2.pdf]

Supplementary table 1: Potential genes and their related pathways

Pathway	Genes	Enrichment FDR	nGenes
Path:oas01521 EGFR tyrosine kinase inhibitor resistance	STAT3, BCL2L11, PDGFRA, TGFA, PIK3CB, SHC4, JAK1, JAK2, PDGFD, AKT3, PIK3R1, PDGFRB, IGF1R	2.68E-08	13
Path:oas03320 PPAR signaling pathway	SCD5, PLIN1, FABP4, ME1, FABP3, SLC27A2, ACSL1, ADIPOQ, PPARD, CPT2, FABP7, SCD, LPL	2.68E-08	13
Path:oas04920 Adipocytokine signaling pathway	PRKAA2, STAT3, PRKAA1, ACSL1, ADIPOQ, JAK2, AKT3, POMC, LEPR, PPARGC1A, LEP	5.53E-07	11
Path:oas04935 Growth hormone synthesis secretion and action	STAT3, PLCB1, ADCY6, PIK3CB, SHC4, JAK2, SOCS2,, CACNA1C, AKT3, PIK3R1, CREB1, SSTR1, SSTR5, GH, GHR	2.76E-08	15
Path:oas04933 AGE-RAGE signaling pathway in diabetic complications	FN1, STAT3, PLCB1, PIK3CB COL4A6, JAK2, AKT3, PIK3R1, TGFB1, COL1A1, TGFBR2, TGFB2, TGFB3,	4.93E-07	13
Path:oas04152 AMPK signaling pathway	SCD5, PRKAA2, LIPE, PRKAA1, PIK3CB, ADIPOQ, AKT3, PIK3R1, CREB1, SCD, ACACA, LEPR, PPARGC1A, INSR, IGF1R	6.94E-08	15
Path:oas04931 Insulin resistance	PRKAA2, STAT3, PRKAA1, OGT, SLC27A2, PIK3CB, GFPT1, AKT3, PIK3R1, PPARGC1B, CREB1, PPARGC1A, INSR	7.18E-07	13
Path:oas04550 Signaling pathways regulating pluripotency of stem cells	STAT3, WNT16, PIK3CB, JAK1, MEIS1, JAK2, AKT3, PIK3R1, KAT6A, FZD6, MYF5, BMPR1B, BMP4, IGF1R,	4.93E-07	14
Path:oas04068 FoxO signaling pathway	PRKAA2, STAT3, BCL2L11, PRKAA1, PIK3CB, SETD7, AKT3, PIK3R1, TGFB1, INSR, IGF1R, TGFBR2, TGFB2 TGFB3	5.53E-07	14
Path:oas04371 Apelin signaling pathway	PRKAA2 PLIN1 LIPE MYL2 PRKAA1 PLCB1 ADCY6 MRAS RYR3 MYLK3 AKT3 SPP1 PPARGC1A UCP1	6.41E-07	14
Path:oas04510 Focal adhesion	MYL2 FN1 ROCK2 ITGA11 PDGFRA VAV1 PIK3CB SHC4 PAK1 COL4A6 MYLK3 PDGFD MYL10 TNC AKT3 PIK3R1 ACTN1 PDGFRB SPP1 COL1A1 IGF1R	3.06E-09	21
Path:oas04932 Non-alcoholic fatty liver disease	PRKAA2 BCL2L11 CYP2E1 PRKAA1 MAP3K11 PIK3CB ADIPOQ AKT3 MAP3K5 PIK3R1 LEPR TGFB1 INSR LEP	1.36E-06	14

Path:oas04810 Reg. of actin cytoskeleton	MYL2 FN1 ROCK2 ITGA11 PDGFRA VAV1 PIK3CB BAIAP2 MRAS PAK1 MYLK3 MYH11 FGF9 PDGFD MYL10 BDKRB2 PIK3R1 ACTN1 PDGFRB	4.63E-08	19
Path:oas05205 Proteoglycans in cancer	FN1 STAT3 WNT16 ROCK2 VAV1 PIK3CB MRAS PAK1 PTCH1 AKT3 CAMK2B PIK3R1 FZD6 TGFB1 COL1A1 IGF1R TGFB2	4.51E-07	17
Path:oas04015 Rap1 signaling pathway	PLCB1 RAP1GAP ADCY6 PDGFRA VAV1 PIK3CB CSF1R MRAS FGF9 PDGFD MAGI1 AKT3 PIK3R1 PDGFRB INSR IGF1R	1.77E-06	16
Path:oas04010 MAPK signaling pathway	PDGFRA TGFA MAP3K11 CSF1R MRAS SRF PAK1 HSPB1 CACNA1C FGF9 PDGFD AKT3 MAP3K14 MAP3K5 PDGFRB CACNA2D1 TGFB1 INSR IGF1R TGFBR2 TGFB2 TGFB3	2.68E-08	22
Path:oas04024 cAMP signaling pathway	LIPE ROCK2 ADCY6 VAV1 PIK3CB PAK1 PTCH1 PDE3A CACNA1C HTR4 AKT3 CAMK2B PIK3R1 CREB1 SSTR1 SSTR5 POMC TSHR	5.53E-07	18
Path:oas04151 PI3K-Akt signaling pathway	PRKAA2 FN1 BCL2L11 PRKAA1 ITGA11 PDGFRA TGFA PIK3CB CSF1R JAK1 COL4A6 JAK2 FGF9 PDGFD TNC MAGI1 AKT3 PIK3R1 PDGFRB SPP1 CREB1 PRL GH GHR INSR COL1A1 IGF1R	3.06E-09	27
Path:oas04060 Cytokine-cytokine receptor interaction	TNFSF8 CSF1R ACVRL1 GDF2 GDF10 BMP2 BMP6 LEPR PRL GH GHR TGFB1 GDF-8 BMPR1B BMP4 LEP TGFBR2 TGFB2 TGFB3	1.02E-06	19
Path:oas05200 Pathways in cancer	FN1, STAT3, BCL2L11, WNT16, PLCB1, ROCK2, ADCY6, PDGFRA, TGFA, PIK3CB, CSF1R, JAK1, PTCH1, COL4A6, JAK2, FGF9, PPARD, BMP2, AKT3, CAMK2B, BDKRB2, PIK3R1, FZD6, PDGFRB, CTNNA3, TGFB1, BMP4, IGF1R, TGFBR2, TGFB2, TGFB3	2.5E-08	31

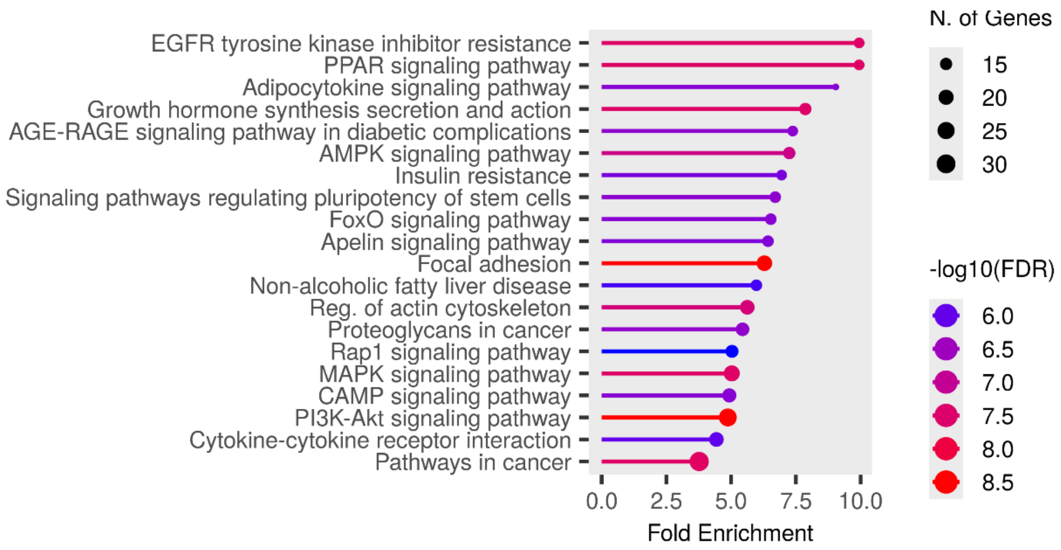

Supplementary figure 1: KEGG pathways regulated by genes selected for our review

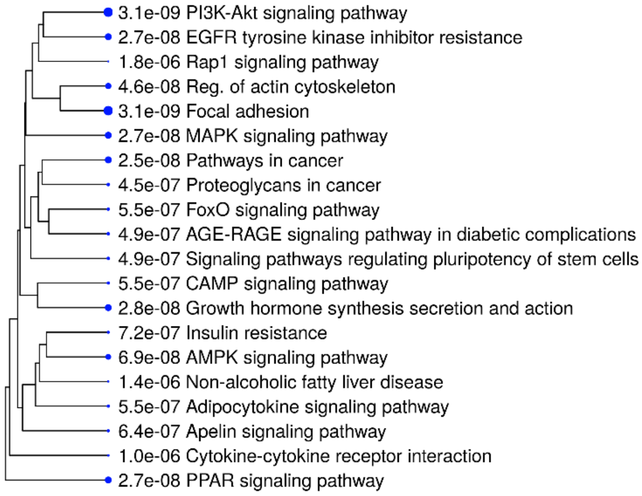

Supplementary Figure 2: Tree for various pathways regulated by genes selected for our review.
